# Shotgun proteomics of quinoa seeds reveals chitinases enrichment under rainfed conditions

**DOI:** 10.1038/s41598-023-32114-5

**Published:** 2023-03-27

**Authors:** Laura Poza-Viejo, Miguel Redondo-Nieto, Javier Matías, Sara Granado-Rodríguez, Isaac Maestro-Gaitán, Verónica Cruz, Enrique Olmos, Luis Bolaños, Maria Reguera

**Affiliations:** 1 Department of Biology, Universidad Autónoma de Madrid, Madrid, Spain; 2 Centro de Investigaciones Científicas y Tecnológicas de Extremadura (CICYTEX), Guadajira, Spain; 3 Department of Abiotic Stress and Plant Pathology, Centro de Edafología y Biología Aplicada del Segura (CEBAS-CSIC), Murcia, Spain

**Keywords:** Plant sciences, Plant stress responses, Abiotic, Drought

## Abstract

Quinoa is an Andean crop whose cultivation has been extended to many different parts of the world in the last decade. It shows a great capacity for adaptation to diverse climate conditions, including environmental stressors, and, moreover, the seeds are very nutritious in part due to their high protein content, which is rich in essential amino acids. They are gluten-free seeds and contain good amounts of other nutrients such as unsaturated fatty acids, vitamins, or minerals. Also, the use of quinoa hydrolysates and peptides has been linked to numerous health benefits. Altogether, these aspects have situated quinoa as a crop able to contribute to food security worldwide. Aiming to deepen our understanding of the protein quality and function of quinoa seeds and how they can vary when this crop is subjected to water-limiting conditions, a shotgun proteomics analysis was performed to obtain the proteomes of quinoa seeds harvested from two different water regimes in the field: rainfed and irrigated conditions. Differentially increased levels of proteins determined in seeds from each field condition were analysed, and the enrichment of chitinase-related proteins in seeds harvested from rainfed conditions was found. These proteins are described as pathogen-related proteins and can be accumulated under abiotic stress. Thus, our findings suggest that chitinase-like proteins in quinoa seeds can be potential biomarkers of drought. Also, this study points to the need for further research to unveil their role in conferring tolerance when coping with water-deficient conditions.

## Introduction

*Chenopodium quinoa* Willd., commonly known as quinoa, is an allotetraploid species (2n = 4x = 36) belonging to the Amaranthaceae family and taxonomically related to beet, spinach, and amaranth^1^. The quinoa genome was recently sequenced enabling a better genomic understanding of this underutilized crop, which possesses a huge genetic diversity (with more than 6000 accessions described) linked to a great capacity for adaptation to a wide variety of environments (including those with high salinity or low water supply) ^2–6^ . In fact, quinoa has emerged as a promising crop whose cultivation has been expanded from its traditional agronomical areas, located in the Andean region, to more than 120 countries with very different climatic conditions, including Spain, France, Morocco, India or Pakistan, although Bolivia and Peru are still the largest producers ^7–13^. Furthermore, quinoa seeds have a remarkable nutritional profile with a high-quality protein composition that provide all the essential amino acids (including the most limiting amino acids in cereals and pulses, which are lysine and methionine, respectively) ^14^. The most abundant proteins in quinoa seeds are the storage proteins 2S albumins and 11S globulins ^15^, this last is described as a specific type in quinoa called chenopodin ^16^. Interestingly, neither prolamins nor other typically present celiac epitopes are found among the quinoa seed profile, giving nutritional value to the seeds as gluten-free food products that can be consumed by celiacs. In addition, quinoa seeds´ hydrolysates and peptides show bioactive properties including antioxidant capacity, antidiabetic, anti-inflammatory, or ACE-related antihypertension activities ^17^. Besides, quinoa seeds also provide polyunsaturated fatty acids, dietary fiber, minerals, and vitamins ^18–20^.

On the other hand, within the current climate context, extensive cultivation areas are expected to suffer from long drought episodes, especially those located in arid or semi-arid regions, such as the Mediterranean region ^21–23^. This, together with the high global demand for food and feed for livestock, requires the selection of climate-resilient and nutritious crops, such as quinoa, which can contribute to global food security ^23^.

Understanding how plants perceive abiotic factors and adapt to adverse environmental conditions (abiotic stresses) is crucial to dealing with environmental and food future scenarios. Plant responses to abiotic stresses comprise complex molecular networks (at transcriptomic, proteomic, and metabolomic levels) that result in morphological, physiological, and molecular adjustments that can lead to protection mechanisms for ensuring plant adaptation and survival under environmental constraints ^24,25^. The extent to which these responses can cause molecular changes usually depends on the type of stress (or the combination of stressors), the duration, and the intensity ^25,26^. In line with these findings, large genomes, with high gene copy number, redundancy, and diversification of gene functions, as occurs in quinoa, confer plasticity to the plant genome architecture, which may contribute to dealing with unfavourable environmental conditions ^25,27,28^.

Eventually, plant strategies can converge in the use of the same protein families to face different stresses and diversify individual functions to respond to certain conditions ^25^. In this regard, plant chitinases conform large gene families, which are expressed under different biotic but also abiotic stresses ^29^. Although plant chitinases are the major and best-characterized pathogen-related (PR) proteins due to their hydrolase activity that enables them to cleave chitin coming from arthropods or fungi, they are also involved in abiotic stress signalling, functioning at different stages of plant development ^29,30^. More specifically, chitinases hydrolyse ß-1,4 bonds that link long-chain polymers of N-acetyl-D-glucosamine, which form chitin´s structure, the second most abundant biopolymer in nature after cellulose ^31^. This cleavage generates small lipo-chito-oligosaccharides (LCOs), which can act as plant resistance elicitors under biotic and abiotic stress in plants, although their functions are still not well characterized ^32^.

Plant chitinases are generally classified into six classes (class I–class VI) based on their genomic sequence and are divided into Glycosyl Hydrolase family 18 (GH18) or Glycosyl Hydrolase family 19 (GH19), depending on their characteristic catalytic domain ^33,34^. Both families have evolved from different ancestral genes, thus, their genomic sequences and 3D protein structures are strongly different ^35^. GH18 chitinases (classes III and V) have typically enzymatic triose-phosphate isomerase (TIM)-barrel fold structure, while GH19 (classes I, II, IV, and VI) have mainly helicoidal protein structure. Also, GH19s are pretty similar to other catalytic enzymes such as chitosanases and lysozymes ^36,37^. Besides, chitinases from class I GH19 possess a chitin-binding domain (ChtBD) at the N-terminal region ^38^. Plant chitinases are usually targeted to the vacuolar compartment or are secreted to the apoplast and are expressed in a tissue-specific manner along the plant ^31^.

Beyond catalytic active chitinases, a large number of genes transcribing chitinase-like proteins (CLPs) are described along plant genomes. CLPs are “inactive” chitinases that share a strong genomic sequence and structure similarity to GH18 or GH19 chitinases. However, they have lost their catalytic activity or their ChtBD, thus providing a source of functional diversification as emerging enzymes able to bind other polysaccharides and/or new catalytic activities hydrolysing diverse substrates ^33^.

Previous quinoa proteomic profiles have been published during the last years describing a discrete number of proteins accumulated in quinoa seeds. However, the lack of accurate genome annotation or proteome information for *C. quinoa* greatly limited the outcomes of these studies. Thus, Capriotti and collaborators ^39^ were only able to identify four specific proteins accumulated in quinoa seeds. In 2019, Burrieza and collaborators ^40^ improved quinoa seed proteomic research utilizing the sequenced genome of the crop ^2–4^, identifying novel seed storage proteins of quinoa which contribute to the characteristic high-lysine content of the seeds. Recently, a descriptive proteomic study identified a total of 1211 seed proteins among four commercial quinoa varieties ^41^. However, none of the studies mentioned above have analysed the impact of abiotic stress on changing the proteomic profile of quinoa seeds.

Here, aiming at analysing the quinoa seed proteome by using a shotgun proteomic approach, we evaluated changes associated with water limitation (rainfed conditions) when compared to full irrigation (irrigated conditions) in quinoa seed samples obtained from the field. In this regard, as far as we know, we report here the most complete quinoa seed proteome to date, finding putative quinoa seed chitinases as an accumulated protein family in quinoa seeds under water limitation. Overall, our data highlight the potential role of chitinases in water stress responses in quinoa and the possibility of using this group of proteins as water stress biomarkers, which can be useful for quinoa breeding programmes and crop improvement strategies.

## Results and discussion

### Proteomic analysis in quinoa seeds harvested from irrigated and rainfed conditions

**Figure 1:**
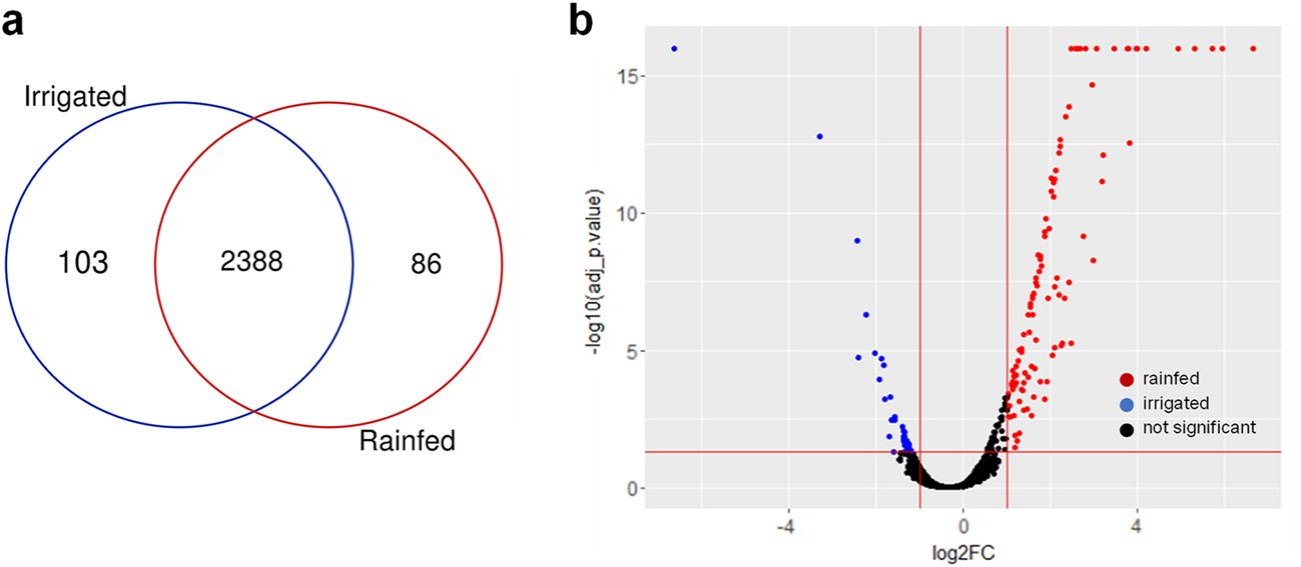
Total proteins quantified in quinoa seeds harvested from irrigated and rainfed conditions. (**a**) From the 2577 identified proteins in quinoa seeds, 103 appeared, exclusively in seeds harvested from irrigated conditions and 86 appeared exclusively in seeds harvested from rainfed conditions. (**b**) Volcano plot representing all the identified proteins in seeds harvested from irrigated and rainfed conditions. Different colours show two-fold differentially abundant proteins for each condition (| log_2_FC ≥ 1 |, *p-adj* ≤ *0.05*; n = 3). Red dots: rainfed conditions; blue dots: irrigated conditions; black dots: no statistically significant accumulated proteins.

In this study, seed protein extracts harvested from quinoa plants grown in the field in rainfed and irrigated conditions were analyzed to identify, quantify, and estimate protein abundance, and to compare protein enrichment between the two water regimes. After raw data collection, Proteome Discoverer 2.4 (Thermo Fisher Scientific, Massachusetts, United States) was used to assign peptides utilizing *C. quinoa* v1.0 proteome dataset available at UniProt database (https://www.uniport.org/proteomes/UP000596660). The total detected proteins were filtered at a *p-adj* < *0.05*, obtaining a total number of 2577 proteins identified in seeds harvested from irrigated and rainfed conditions (Fig. 1A and Supplementary Tables S1 and S2). The proportion of proteins on the basis of single-matching peptides was 17.8% (458 proteins). Protein abundance in the samples was analyzed by principal component analysis (PCA) appearing in two separate groups, which corresponded to each water regimen, irrigated or rainfed conditions (Supplementary Fig. S2). When comparing these values with the proteomic results obtained by Galindo-Luján and collaborators ^41^ , using quinoa seeds from four different commercial varieties, in which 1211 proteins were identified by using LC–MS/MS, our current analysis yielded a significantly higher number of proteins. Besides, from the total of 2577 proteins identified, 2388 proteins (93% of the total) appeared in both conditions, 103 proteins (4% of the total) were exclusively found in seeds harvested from irrigated conditions, and 86 proteins (3% of the total) were exclusively identified in seeds harvested from rainfed conditions (Fig. 1A and Supplementary Table S2). Thus, although a great number of proteins were found in both water conditions, proteins exclusively represented in each condition depicted a low percentage from the total.

To determine whether there were quantitative differences regarding protein abundance in seeds harvested from irrigated or rainfed conditions, a differential statistical analysis of the proteins found in rainfed compared to irrigated conditions was performed to search for quantitative changes. As seen in Fig. 1B, a higher abundance of proteins in seeds harvested from rainfed conditions (196 proteins) was found compared to that number in seeds harvested from irrigated conditions (142 proteins). Accordingly, the dot´s distribution in the volcano plot appeared to shift to the right side, which represents protein overabundance in rainfed conditions (log2(FC) ≥ 1).

### Seed Storage Proteins (SSP) and other seed-related proteins

Among the shared proteins obtained from our study in both water conditions, different seed storage proteins (SSP) were found, including the 2S albumins and two 11S globulins, also known as chenopodins (Table 1). Both classes of proteins are the major storage proteins found in quinoa seeds, as described in previous works ^40–42^. Interestingly, in addition to presenting substantial amounts of essential amino acids in their composition, chenopodins have been recently linked to anti-inflammatory properties in mice ^43^. Alternatively, the SSP 2S albumin, one of the major protein classes found in quinoa seeds, as first described by Brinegar and collaborators ^44^ possesses significant contents of sulfur amino acids such as cysteine and also, histidine, and arginine. Both types of proteins have been identified in quinoa seed samples using new approaches based on shotgun proteomics^41^ and this work). In addition, several 7S globulins and 13S globulins (Table 1) appeared in the seed protein samples obtained from rainfed and irrigated conditions and were also present in previous proteomic analyses carried out by Burrieza and collaborators ^40^. Since these SSP were consistently found in seeds obtained from different quinoa varieties, this, and previous studies, suggest a homogeneous and conserved distribution of SSP among different quinoa cultivars. Furthermore, our results confirm that the presence or abundance of these SSPs does not vary depending on the water regime, rainfed or irrigated conditions, at least in seeds harvested from the quinoa cultivar used in this study (Supplementary Table S2).

**Table 1:** SSPs and other characteristic seed-related proteins simultaneously found in seeds harvested from irrigated and rainfed conditions.

*C. quinoa* ID	Description
AUR62015663-RA	2S albumin
AUR62020540-RA	2S albumin
AUR62011869-RA	11S globulin (chenopodin)
AUR62024712-RA	11S globulin (chenopodin)
AUR62028591-RA	7S globulin
AUR62032318-RA	7S globulin
AUR62034727-RA	7S globulin
AUR62033661-RA	7S globulin
AUR62015569-RA	13S globulin
AUR62022853-RA	Seed oil body oleosin
AUR62012221-RA	Seed oil body oleosin
AUR62040213-RA	Seed oil body oleosin
AUR62008167-RA	Seed oil body oleosin
AUR62036943-RA	Seed oil body oleosin
AUR62002243-RA	Seed oil body oleosin
AUR62004102-RA	Dehydrin family protein
AUR62011287-RA	Late Embryogenesis Abundant (LEA) and LEA-related protein
AUR62034707-RA	Late Embryogenesis Abundant (LEA) and LEA-related protein
AUR62043549-RA	Late Embryogenesis Abundant (LEA) and LEA-related protein
AUR62032331-RA	Late Embryogenesis Abundant (LEA) and LEA-related protein
AUR62028605-RA	Late Embryogenesis Abundant (LEA) and LEA-related protein
AUR62028603-RA	Late Embryogenesis Abundant (LEA) and LEA-related protein
AUR62014787-RA	Late Embryogenesis Abundant (LEA) and LEA-related protein
AUR62002497-RA	Late Embryogenesis Abundant (LEA) and LEA-related protein
AUR62023689-RA	Late Embryogenesis Abundant (LEA) and LEA-related protein
AUR62018728-RA	Late Embryogenesis Abundant (LEA) and LEA-related protein
AUR62007271-RA	Late Embryogenesis Abundant (LEA) and LEA-related protein
AUR62014840-RA	Late Embryogenesis Abundant (LEA) and LEA-related protein
AUR62017037-RA	Late Embryogenesis Abundant (LEA) and LEA-related protein
AUR62011567-RA	Late Embryogenesis Abundant (LEA) and LEA-related protein
AUR62022650-RA	Late Embryogenesis Abundant (LEA) and LEA-related protein
AUR62037387-RA	Late Embryogenesis Abundant (LEA) and LEA-related protein
AUR62042308-RA	Late Embryogenesis Abundant (LEA) and LEA-related protein
AUR62002551-RA	Late Embryogenesis Abundant (LEA) and LEA-related protein
AUR62029965-RA	Late Embryogenesis Abundant (LEA) and LEA-related protein
AUR62012039-RA	Late Embryogenesis Abundant (LEA) and LEA-related protein
AUR62022623-RA	Late Embryogenesis Abundant (LEA) and LEA-related protein
AUR62032329-RA	Seed Maturation family protein
AUR62028604-RA	Seed Maturation family protein
AUR62037914-RA	Embryonic Cell LEA-related protein
AUR62040165-RA	Embryonic Cell LEA-related protein

Besides, seed oil body oleosins, a dehydrin family protein, late embryogenesis abundant (LEA) and LEA-related proteins, seed maturation family proteins, and embryonic cell LEA-related proteins were found among the identified seed proteins obtained from both irrigated and rainfed conditions (Table 1), all of them related to characteristic desiccation and maturation processes occurring in seeds ^45,46^.

### Biological and functional significance of irrigated and rainfed quinoa seeds´ proteomic profiles

**Figure 2:**
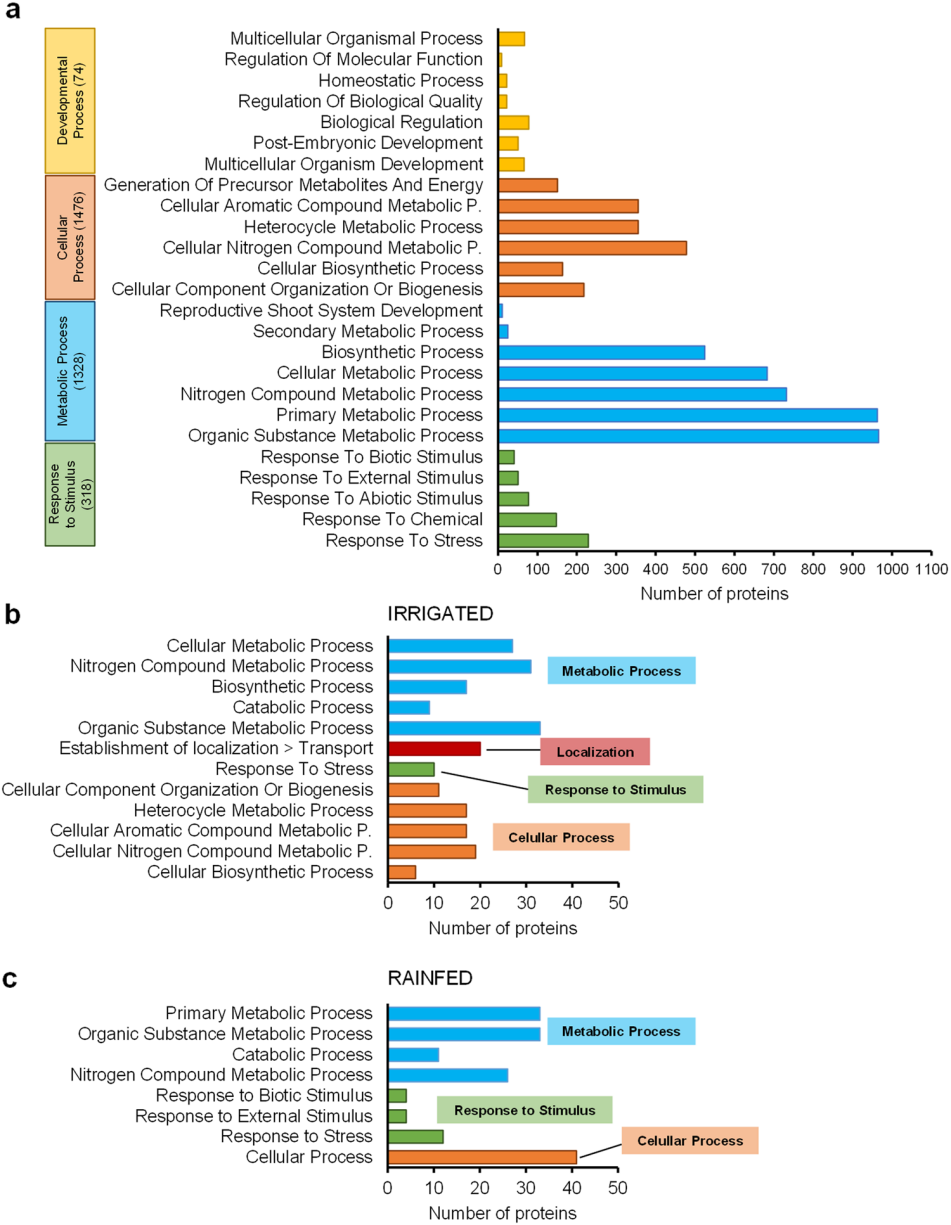
Seed proteins harvested from irrigated and rainfed conditions classified by gene ontology (GO) terms related to Biological Process (BP). (**a**) From the total of 2388 proteins quantified which were found in both conditions, 1960 were associated to BP-GO terms. In the graphs, the widest GO terms related to BP identified for these 1960 proteins were represented. (**b**) From the 103 proteins quantified exclusively under irrigated conditions, 81 were associated with BP-GO terms. (**c**) From the 86 proteins quantified exclusively under rainfed conditions, 81 were associated with BP-GO terms.

In order to decipher the biological functions attributed to the proteins identified in quinoa seeds harvested from both irrigated and rainfed conditions, a gene ontology (GO) analysis was performed. Shared and exclusive protein enrichment was analyzed to evaluate the Biological Process GO terms association (Fig. 2).

#### Biological process GO terms enrichment in proteins annotated simultaneously in seeds from irrigated and rainfed conditions

A total of 1960 proteins out of the 2388 shared proteins previously identified were associated with Biological Process (BP) GO terms (Fig. 2A). A large number of proteins were assigned to two main BP categories: *metabolic process* (GO:0008152; 1328 proteins) and *cellular process* (GO:0009987; 1476 proteins). Among them, there was a great number of GO terms related to the *primary* (GO:0044238; 963 proteins) and *organic substance metabolic process* (GO:0071704; 966 proteins), metabolic processes of nitrogenous compounds (*nitrogen compound metabolic process* GO:0006807, 732 proteins; *cellular nitrogen compound metabolic process* (GO:0034641, 478 proteins), *cellular metabolic process* (GO:0044237, 683 proteins), *biosynthetic process* (GO:0009058, 525 proteins), *cellular aromatic compound metabolic process* (GO:0006725; 356 proteins), and to the *heterocycle metabolic process* (GO:0046483; 356 proteins) (Fig. 2A). These categories were followed, in protein number, by the BP category *response to stimulus* (GO:0050896; 318 proteins) in which the *response to stress* (GO:0006950; 229 proteins) was the one presenting a larger protein number (Fig. 2A). The category *developmental process* (GO:0032502; 74 proteins) only involved GO terms related to *anatomical structure development* (GO:0048856; 73 proteins), detailed in Fig. 2A.

#### Biological Process GO terms enrichment of seed proteins from plants harvested from irrigated conditions

On one hand, a total of 81 proteins out of the 103 proteins exclusively found in irrigated conditions were associated to BP-GO terms (Fig. 2B). BP GO categories such as *cellular biosynthetic process* (GO:0044249, 6 proteins), *cellular nitrogen compound metabolic process* (GO:0034641; 19 proteins), *cellular aromatic metabolic process* (GO:0006725; 17 proteins) and *heterocycle metabolic process* (GO:0046483; 17 proteins) belonging to *cellular metabolic process* (GO:0044237; 27 proteins), and also included in *cellular process* GO term (GO:0009987; 63 proteins, not detailed, as they were the same categories), were categories exclusively present among proteins from seeds harvested from irrigated conditions. Also, metabolic processes such as *organic substance biosynthetic process* (GO:1901576; 6 proteins) and *cellular biosynthetic process* (GO:0044249; 6 proteins) were unique categories from samples obtained from this water condition, and GO categories related to *localization* (GO:0051179; 20 proteins), *establishment of localization* (GO:0051234; 20 proteins) and *transport* (GO:0006810; 20 proteins) as well (Fig. 2B).

#### Biological Process GO terms enrichment of seed proteins from plants harvested from rainfed conditions

On the other hand, within the 86 proteins exclusively found in rainfed conditions, 81 were associated to BP-GO terms (Fig. 2C). We remarkably found the subcategories *carbohydrate metabolic process* (GO:0005975; 7 proteins, not shown) and *protein metabolic process* (GO:0019538; 18 proteins, not shown), belonging to *primary metabolic process* (GO:0044238; 33 proteins), enriched in seeds under rainfed condition. Some proteins were assigned to the category *response to stimulus* (GO:0050896; 12 proteins) that fell out into the subcategories *response to stress* (GO:0006950; 12 proteins), *biotic stimulus* (GO:0009607; 4 proteins) and *external stimulus* (GO:0009605; 4 proteins), these last two categories only found under water limiting conditions (Fig. 2C).

Although proteins listed in the mentioned above sections were exclusive to each water condition, some of them fell into the same BP category (including GO terms assigned to *nitrogen compound metabolic process*, *catabolic process*, *organic substance metabolic process*, and *response to stress*). This result might imply that, despite being different proteins, they might share functionality (*eg*. AUR62024052 annotated as a peroxidase from rainfed seeds and AUR62013045 annotated as L-ascorbate peroxidase 3, were both classified into the *catabolic process* term, GO:0009056, respectively); or they can also belong to the same BP category without sharing similarities in their function (*eg*. AUR62006492 annotated as a mitogen-activated protein kinase 3 (MPK3) from rainfed seeds, and AUR62032691 annotated as a glutamate dehydrogenase B (GDHB), were both classified into the exclusive rainfed or irrigated *response to stress* term, GO:0006950, respectively) (Supplementary Table S2). Therefore, these results suggest that although differences may appear in the protein that is synthesized, similar or dissimilar cellular or metabolic processes might have concurred.

#### GO terms showed differential enrichment of antioxidant-related proteins in seed proteomes from plants harvested from rainfed conditions

**Figure 3:**
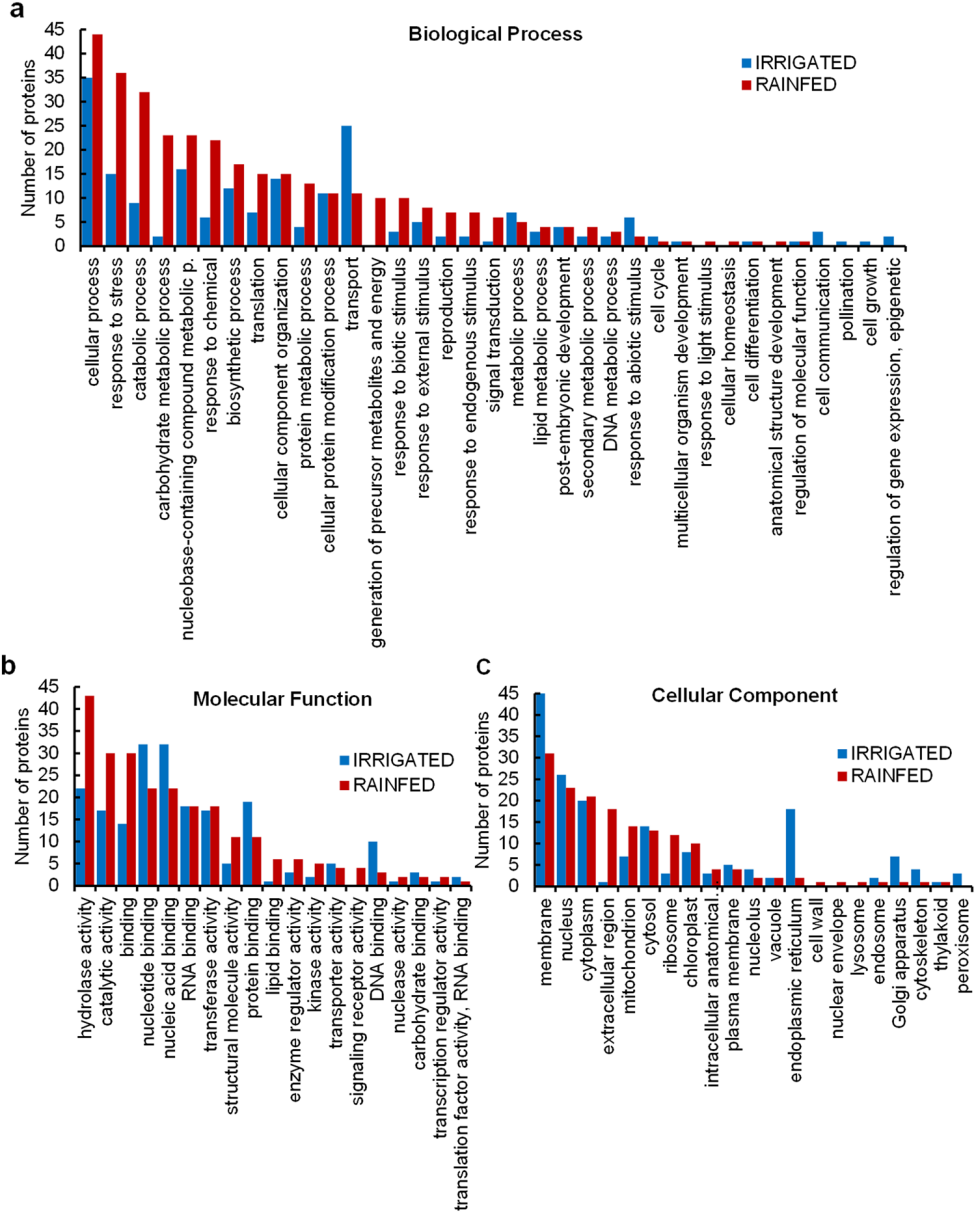
Gene ontology (GO) annotation of differentially accumulated proteins in seeds harvested from irrigated and rainfed conditions. The graph represents the number of statistically significant abundant proteins in seeds harvested from rainfed and irrigated conditions (| log_2_FC ≥ 1 |, *p-adj* ≤ *0.05*; n = 3) assigned to (**a**), Biological Process (BP) (**b**) Molecular Function (MF) or (**c**), Cellular Component (CC) GO categories. From the total of 196 differentially accumulated proteins in seeds harvested from rainfed compared to irrigated conditions, 170 were assigned to GO terms that belong to the categories Biological Process (BP), Molecular Function (MF), or Cellular Component (CC). Seed proteins from irrigated conditions samples yielded 126 proteins, from a total of 142, that were assigned to BP, MF, and CC-GO terms.

As the number of proteins found exclusively in each condition was limited, to deepen the understanding of possible mechanisms related to altered protein profile in seeds harvested from rainfed conditions, GO terms were assigned to proteins that showed statistically larger abundance in seeds harvested from rainfed conditions compared to irrigated conditions (Supplementary Tables S3 and S4). The GO analysis (including Biological Process, BP, Molecular Function, MF and Cellular Component, CC, terms) revealed interesting differences among water conditions (Fig. 3A–C and Supplementary Figs. S4-S9). Protein enrichment under irrigated conditions was found related to *transport* (GO:0006810) regarding BP-GO terms (Fig. 3A and Supplementary Fig. S4); and *protein binding* (GO:0005515), *nucleotide binding* (GO:0000166), *nucleic acid binding* (GO:0003676) and *DNA binding* (GO:0003677) within the enriched MF-GO terms (Fig. 3B and Supplementary Fig. S5). On the other hand, BP-GO terms enrichment in seeds harvested from rainfed conditions presented a large number of proteins related to *response to stress* (GO:0006950), *response to biotic stimulus* (GO:0009607), *response to external* (GO:0009605) and *endogenous stimulus* (GO:0009719), and *response to chemicals* (GO:0042221) (Fig. 3A and Supplementary Fig. S6). Along with this striking representation of proteins responding to stress and stimuli, a remarkable number of them were also related to *catabolic process* (GO:0009056), *carbohydrate metabolism* (GO:0005975), and *protein metabolic process* (GO:0019538). This enrichment coincided with MF-GO terms involved in *binding* (GO:0005488), *hydrolase activity* (GO:0016787), and *catalytic activity* (GO:0003824) (Fig. 3B and Supplementary Fig. S7), standing out the importance of catalytic mechanisms triggered under rainfed conditions. These results are also supported by the enrichment of proteins under this condition that fell into the *generation of precursor, metabolites, and energy* (GO:0006091) BP term.

A characteristic systemic drought-response mechanism in quinoa is the synthesis of reactive oxygen species (ROS) scavengers, together with the accumulation of osmolytes and antioxidants. Particularly, those synthesized in the ornithine and raffinose pathways but also the accumulation of soluble sugars and proline, which also contribute to the cellular osmotic adjustment (reviewed in ^27,47^). This enhanced accumulation of ROS detoxification enzymes has been recently described in 4-week-old quinoa seedlings subjected to salinity stress ^48^. In line with these findings, numerous antioxidant enzymes were accumulated in seeds harvested from rainfed conditions and were categorized into *catabolic process* BP-GO term, such as L-ascorbate peroxidases (AUR62044027-RA, AUR62003342-RA), peroxidase (POD) (AUR62024052-RA), cytochrome C peroxidase (AUR62003343-RA), peroxidase C1C (AUR62026666-RA), peroxidase 4 (AUR62012343-RA, AUR62009723-RA), cathepsin B (AUR62001249-RA), plastidial pyruvate kinase 2 (AUR62021072-RA), peroxiredoxin-2E (AUR62037884-RA), fructose-bisphosphate aldolases 3 (AUR62033531-RA, AUR62028580-RA), glutathione S-transferase (GST) (AUR62008599-RA) and Cu/Zn superoxide dismutase (SOD) (AUR62000929-RA). Under water deficiency, plant tissues accumulate ROS ^45,49^. As a consequence, plants respond by triggering ROS scavenging systems to avoid the oxidation of biomolecules that could hinder cellular homeostasis ^49^. In our experiment, seeds from quinoa grown under rainfed conditions accumulated these types of enzymes. Similarly, other crops such as maize induce ROS scavenging enzymes (such as SOD, POD, and GST) as an early response mechanism to drought ^50^. Moreover, ROS molecules play a fine-tuning role in regulating seed dormancy release and germination, although they could trigger seed deterioration when produced in high concentrations causing DNA/RNA damage, lipid peroxidation, or protein carbamylation (reviewed by ^51^). Nonetheless, the regulatory mechanisms controlling ROS balance under stress are still not well defined, although one can speculate that seeds promote dormancy to avoid tissue damage as a result of ROS accumulation, while the activation of ROS scavenging systems can be an effective response to reduce ROS concentrations when reaching extremely harmful levels.

Other enzymes differentially present in quinoa seeds harvested from rainfed conditions were the aspartic proteinases (AUR62006817-RA, AUR62000476-RA), nicastrin (AUR62040737-RA), and cysteine proteinase inhibitors (AUR62021845-RA, AUR62012808-RA), related to *protein metabolic process* BP-GO term. Moreover, enzymes such as the fructose-bisphosphate aldolase 3 (AUR62033531-RA, AUR62028580-RA), the cytochrome C (AUR62027049–RA, AUR62027048-RA), the plastidial pyruvate kinase 2 (AUR62021072-RA), the NADH dehydrogenase [ubiquinone] 1 beta subcomplex subunit 9 (AUR62010388-RA), the NADP isocitrate dehydrogenase (AUR62002238-RA), and the plastocyanin like domain (AUR62013468-RA, AUR62026803-RA) were accumulated in seeds harvested from rainfed conditions. Overall, these results showed the accumulation of characteristic abiotic stress response proteins, antioxidant enzymes, and proteins involved in energy metabolism. Supporting these results, it was previously observed that desiccation is also able to induce the accumulation of these types of proteins in tea (*Camellia sinensis*) recalcitrant seeds (desiccation-sensitive seeds) in response to redox status alteration ^52^.

Intriguingly, our data revealed a pathogen-related (PR) protein accumulated (and exclusively present) in rainfed conditions, the germin-like protein (GLP) AUR62037551-RA (Supplementary Table S3). GLPs genes are described to be induced in quinoa under *Trichoderma* symbiotic interaction ^53^. However, plant genomes contain a large number of GLPs copies with putative diverse enzymatic activities as SOD or ADP-glucose pyrophosphatase/phosphodiesterase (AGPPase) activities, in addition to their canonical function as oxalate oxidases (OXO) that increase their activity under abiotic stresses in plants ^54^.

In this regard, as previously mentioned, our proteomic study has revealed an enhanced protein accumulation of enzymatic strategies related to ROS scavenging and cellular detoxification alleviation in seeds harvested from rainfed conditions. Therefore, GLP enzymatic activity may contribute to this putative drought-avoidance strategy that quinoa seeds developed under water-deficient conditions.

Besides, the accumulated seed proteins in rainfed conditions were preferentially assigned to CC-GO terms as *extracellular region* (GO:0005576), *mitochondrion* (GO:0005739), and *ribosome* (GO:0005840) (Fig. 3C and Supplementary Fig. S8), where peroxidases and other catabolic enzymes above described for BP and MF-GO terms were also found. Under well-watered conditions, seed proteins that belong to the *membrane* (GO:0016020), *endoplasmic reticulum* (GO:0005783), and *Golgi Apparatus* (GO:0005794) terms were significantly higher (Fig. 3C and Supplementary Fig. S9). According to these findings, the major groups of highly accumulated seed proteins under irrigated conditions were heat shock proteins (AUR62015029-RA, AUR62017485-RA, AUR62014325-RA, AUR62021118-RA, AUR62035682-RA, and AUR62017128-RA) and calnexin homologs (AUR62032201-RA and AUR62036970-RA), among others (Supplementary Table S3). This HSPs´ accumulation under irrigated conditions might favour the cellular defence against pathogens as these proteins have been related with plant immune response ^55^. Interestingly, calnexins are proteins related to endoplasmic reticulum (ER) stress, which can be triggered by abiotic and biotic stresses ^56^. However, the two differentially abundant calnexin homologs in seeds harvested from irrigated conditions were also found in previously published works analysing quinoa seeds not subjected to stress ^41^.

Besides, within the seed proteins presenting increased accumulation in rainfed conditions, we identified a ER stress response protein, the somatic embryogenesis receptor kinase 1 (SERK1), AUR62018453-RA, which has been described as a co-receptor kinase linked to ER-associated degradation (ERAD), induced to alleviate ER stress in plants ^57^ that, in our case, could be induced by drought in seeds.

### Chitinase-related proteins were differentially accumulated in quinoa seeds harvested from rainfed conditions

As previously mentioned, among the most represented GO categories that included highly accumulated proteins in rainfed conditions, we found *hydrolase* and *catalytic activities*, *catabolic process,* and *carbohydrate metabolism* and *response to stress*. When analysing the proteins assigned to those GO terms, the protein family chitinase appeared to be predominant under water-limiting conditions.

Chitinases are chitin hydrolases that are expressed in plants in response to biotic stresses (PR proteins), during plant development, or in response to abiotic stresses ^29^. Seed chitinases seem to play multiple roles in seed germination and seedling establishment as a part of the defence response against microbes ^58^. However, the specific functions that these proteins possess have been little explored.

**Figure 4:**
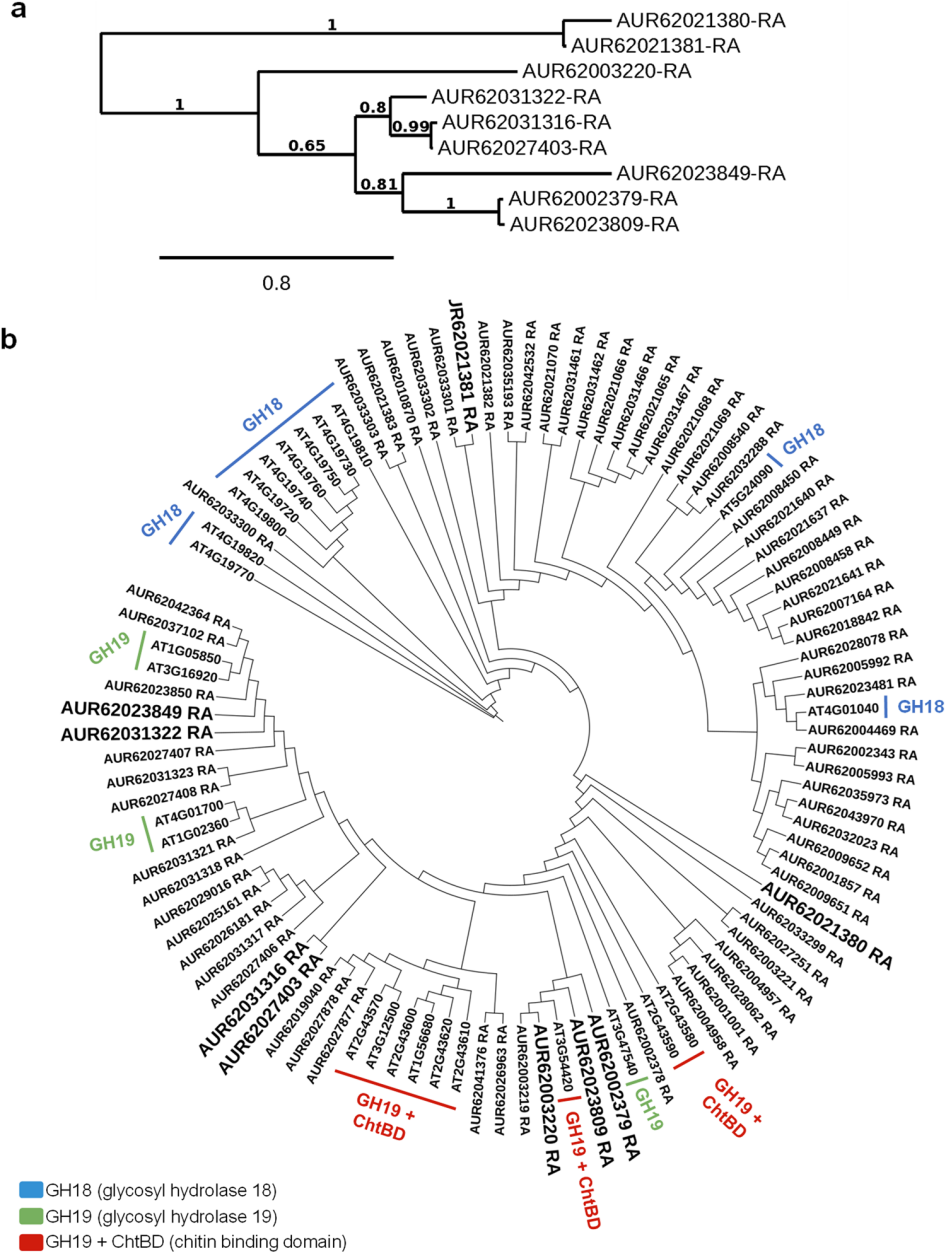
Phylogenetic trees of chitinase-like proteins identified in quinoa. (**a**) Chitinase-like proteins found in seeds harvested from rainfed conditions in *C. quinoa.* (**b**) Phylogenetic tree containing 76 chitinase-like proteins annotated in *C. quinoa* genome v1.0 (Phytozome v13). Their peptide similarity was analysed including the 25 chitinases described in model plant *A. thaliana*, grouped according to their functional domains using NGPhylogeny.fr.

Here, we identified 9 chitinase-related proteins accumulated in seeds harvested from rainfed conditions (Fig. 4A) among the total number of 76 chitinase-related proteins found in *C. quinoa* genome v1.0 (Phytozome v13). Based on their peptide sequences, a phylogenetic tree was obtained including the 25 peptide sequences of *Arabidopsis thaliana* chitinases previously described by Grover and collaborators ^29^ (Fig. 4B).

Plant chitinases are divided into two main families, GH18 and GH19, based on their protein structure, which determines their catabolic activity ^33^. Also, in plants, there are numerous copies of Chitinase-Like Proteins (CLPs) that conserve one of these two types of protein structures. Although some CLPs have lost their chitin-binding domain, they have diversified their catalytic activities and could bind other substrates ^33^. Among the differentially accumulated quinoa chitinases in seeds harvested from rainfed conditions, we found both types, GH18-like (AUR62021380-RA and AUR62021381-RA) and GH19-like (AUR62027403-RA and AUR62023849-RA) chitinases (Table 2). Additionally, a conserved N-terminal Chitin Binding Domain (ChtBD) followed by a GH19-like domain has been described in four of the identified proteins (AUR62002379-RA, AUR62031322-RA, AUR62027403-RA and AUR62023809-RA) resembling typical class I GH19 plant chitinases; and a ChtBD *solo* peptide (AUR62003220-RA) was also found (Table 2).

**Table 2:** Results from NCBI Batch Web CD-search tool for protein domain prediction and *C. quinoa* v1.0 annotation from Phytozome13 (From…to: range of amino acids in the query protein sequence to which the domain model aligns; E-Value: expected value, the statistical significance of the hit as the likelihood the hit was found by chance; Accession: accession number of the hit, cd = conserved domain from NCBI, cl = superfamily cluster; Superfamily: specific accession number of the superfamily to which the domain model belongs; Short name: defining name for the conserved domain). Underlined AUR codes show chitinase-related proteins exclusively identified in seeds harvested from rainfed conditions.

Query	Description *C. Quinoa* v1.0	From	To	E-Value	Accession	Superfamily	Short name
AUR62021380-RA	Acidic mammalian chitinase	123	246	2,61E−06	cd02879	cl10447	GH18_plant_chitinase_class_V
AUR62021381-RA	Endochitinase 46	27	355	5,61 E−116	cd02879	cl10447	GH18_plant_chitinase_class_V
		70	351	1,11 E−33	cl34587	–	ChiA superfamily
AUR62002379-RA	Acidic endochitinase SP2	83	282	2,21 E−80	cd00325	cl00222	chitinase_GH19
		83	282	2,21 E−80	cl00222	–	Lyz-like superfamily
		32	59	1,72 E−02	cd00035	cl16916	ChtBD1
AUR62031322-RA	Endochitinase EP3	75	270	6,24 E−77	cd00325	cl00222	chitinase_GH19
		75	270	6,24 E−77	cl00222	–	Lyz-like superfamily
		29	52	1,24 E−02	cd00035	cl16916	ChtBD1
AUR62031316-RA	Basic endochitinase C	47	242	4,98 E−77	cd00325	cl00222	chitinase_GH19
		47	242	4,98 E−77	cl00222	–	Lyz-like superfamily
AUR62027403-RA	Chitinase 4	76	271	5,92 E−76	cd00325	cl00222	chitinase_GH19
		76	271	5,92 E−76	cl00222	–	Lyz-like superfamily
		29	53	2,82 E−02	cd00035	cl16916	ChtBD1
AUR62003220-RA	Antimicrobial peptide 2	31	52	8,46 E−02	cd00035	cl16916	ChtBD1
AUR62023809-RA	Acidic endochitinase SP2	84	283	5,58 E−79	cd00325	cl00222	chitinase_GH19
		84	283	5,58 E−79	cl00222	–	Lyz-like superfamily
		32	59	1,50 E−02	cd00035	cl16916	ChtBD1
AUR62023849-RA	Chitinase 3	10	240	1,67 E−105	cd00325	cl00222	chitinase_GH19
		10	240	7,03 E−132	cl00222	–	Lyz-like superfamily

**Figure 5:**
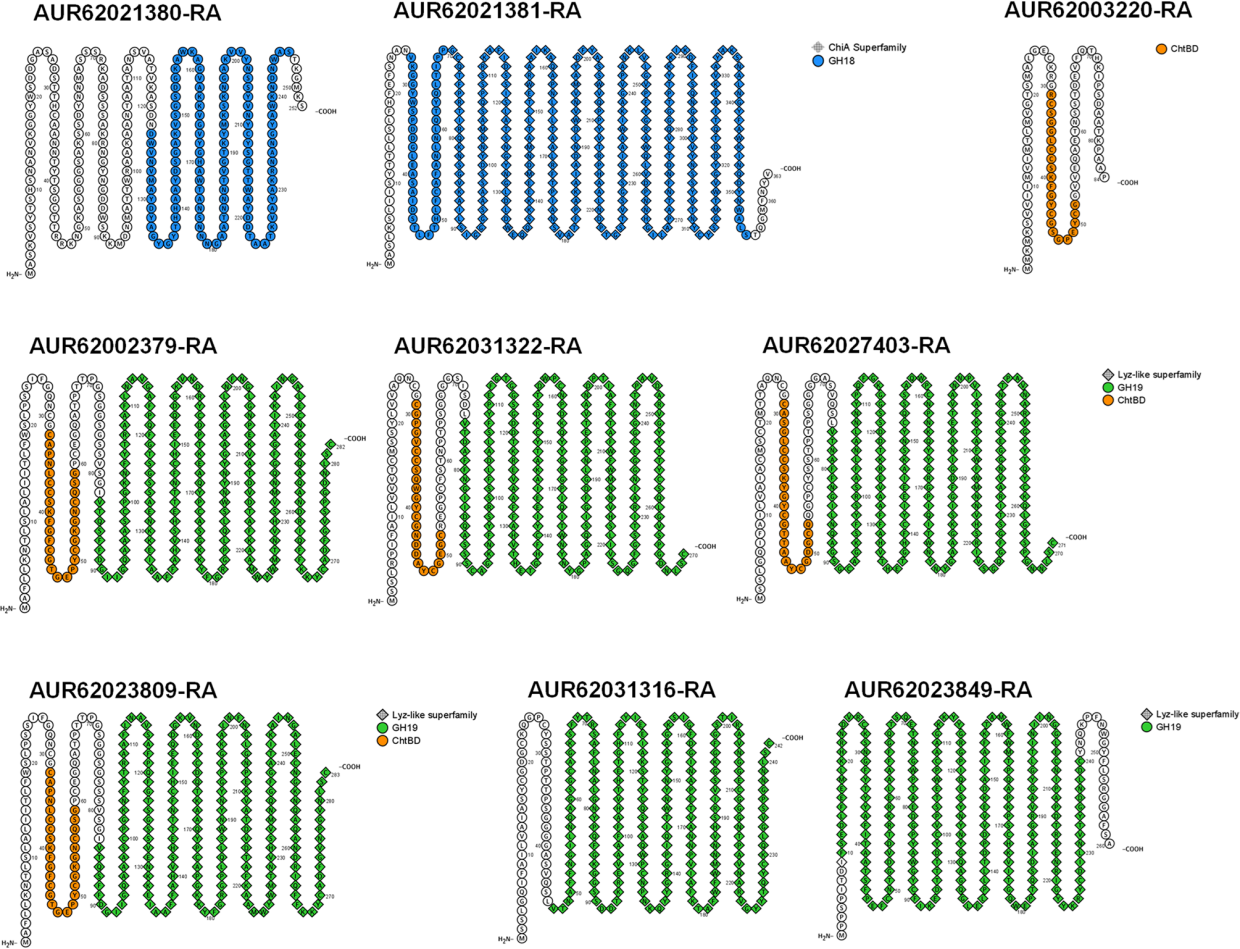
Conserved domain (CD) prediction for the 9 chitinase-related proteins identified in quinoa seeds under rainfed conditions. Representation of predicted conserved domains for the 9 chitinase-related proteins identified in quinoa seeds harvested from rainfed conditions, using Protter (http://wlab.ethz.ch/protter/start/).

In *Oryza sativa* ^59^, *Bryum coronatum* ^60^, and *Picea abies* ^61^, the GH19 chitinase family was the most predominant and well-characterized chitinase family found in these plant species. Also, GH19 chitinases have been reported as the most important family representing seed chitinases ^62^ and, indeed, they were the most abundant chitinase type found in our study (AUR62027403-RA, AUR62023849-RA AUR62002379-RA, AUR62031322-RA, AUR62027403-RA, and AUR62023809-RA) (Fig. 5 and Table 2).

However, from the 9 chitinase-related proteins differentially accumulated in seeds harvested from rainfed conditions, only AUR62021380-RA (GH18-like) and AUR62023849-RA (GH19-like) were exclusively detected under such conditions. This result pointed these two chitinases-like proteins as potential candidates to be used as drought molecular markers for quinoa seeds.

The annotated chitinase-related proteins in *C. quinoa* genome v1.0 were obtained as homologous of the chitinases described in other organisms (Table 2). Moreover, the domain prediction performed in the chitinase-related proteins found in *C. quinoa*, based on the results yielded by the NCBI Batch Web CD-search tool (https://www.ncbi.nlm.nih.gov/Structure/bwrpsb/bwrpsb.cgi) displayed GH18, GH19 and ChtBD domains characteristic of this protein family (Fig. 5 and Table 2), confirming their conserved sequence and their possible role as chitinases or CLPs in response to drought stress in quinoa seeds. In line with this finding, recent results from Rasouli and collaborators ^63^ showed proteome profiles of guard cells in quinoa in response to osmotic stress regulated by ABA signalling. Among the proteins differentially abundant under salinity stress, Rasouli and collaborators ^63^ found an increase in chitinases-related proteins, coinciding with two out of the four chitinases found in seeds harvested from rainfed conditions (AUR62021381-RA GH18-like chitinases and AUR62023809-RA GH19-like chitinases + ChtBD). Moreover, AUR62021381-RA is highly similar to ChiA superfamily chitinases, whose homolog in pepper (*CaChi2*) is able to increase the tolerance to osmotic stress when overexpressed in *A. thaliana* ^64^.

It is worth mentioning that no chitinase-related proteins have been previously detected in shotgun proteomics in quinoa seeds ^40,41^, reinforcing the idea that the differential accumulation of chitinase-related proteins in quinoa seeds appears in response to water constraint since none of the previously published proteomic experiments worked with seed harvested from water stress conditions. Therefore, even though plant chitinases seem to show tissue-specificity, as reported in other plant species such as sugar cane ^65^, similar abiotic stress signalling pathways could occur in different types of cells or tissues, giving rise to the importance of some specific chitinase-related proteins as proteins that participate in signal-transduction networks that operate under abiotic stress.

**Figure 6:**
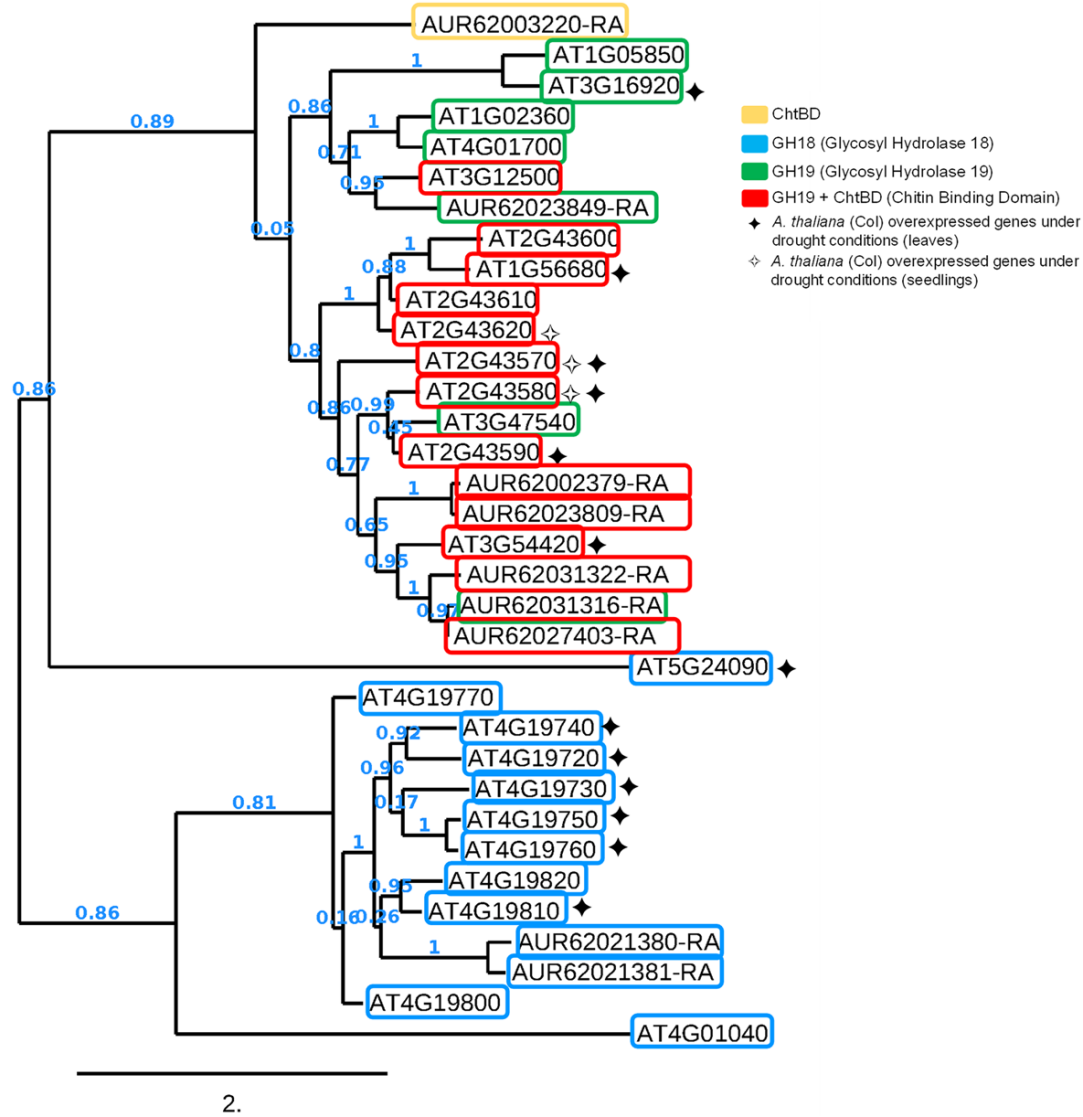
Phylogenetic tree of *A. thaliana* chitinases and quinoa chitinase-related proteins found in seeds under rainfed condition. Twenty-five chitinases have been identified in *A. thaliana* which are mainly divided into three groups based on their catalytic and binding domains. The 9 chitinase-like proteins differentially abundant under rainfed conditions in the quinoa seeds analyzed, showed sequence similarities to protein domains of *A. thaliana* ones. In addition, quinoa chitinase-like proteins were closer to the ones that were highly expressed in microarray data from leaves and seedlings of *A. thaliana* grown under drought conditions from two independent experiments summarized in ^29^.

Interestingly, chitinase-like proteins identified in rainfed samples were grouped according to their functional domains based on their homology with the *A. thaliana* chitinases (Fig. 6). In *A. thaliana*, chitinase transcripts were notably upregulated in seedlings, leaves, shoots and roots subjected to different drought conditions ^29^. These results were also supported by the work performed by Rasheed and collaborators ^66^, in which the chitinase gene *AT2G43570* was highly upregulated under drought stress in shoots and roots. These *A. thaliana* chitinases were closely related to the quinoa chitinase-related proteins detected in rainfed seeds (Fig. 6), suggesting conserved roles in response to drought among this taxonomically distant plant species and in the different tissues analysed. Other studies have shown increments of endochitinase protein abundance during vegetative and flowering stages under drought stress in common bean (*Phaseolus vulgaris* L.) ^67^, similar to the accumulation of diverse endochitinase-like proteins (AUR62021381-RA, AUR62002379-RA, AUR62031322-RA, AUR62031316-RA, AUR62023809-RA) found in quinoa seeds harvested from rainfed conditions (Table 2). In addition, several GH19-like chitinases from Manchurian wild rice (*Zizania latifolia* L.) increased their expression under abiotic stresses ^68^ and the accumulation of plant chitinases was found in roots of barley, corn, pea, soybean, and beans in response to heavy metal toxicity ^69^. Other environmental stresses ^70^ also induced the accumulation of chitinases in agronomically important species such as *Lycopersicon chilense* ^71^, bromegrass ^72^, or blueberry ^73^. Likewise, the overexpression of the *CHITINASE 2* (*LcCHI2*) from wheatgrass (*Leymus chinensis*) in transgenic tobacco and maize plants showed increased tolerance to saline-alkali stress ^74^ and tea (*C. sinensis*) desiccation-sensitive (recalcitrant) seeds accumulate a homolog of the chitinase-related AUR62023849-RA that was found in quinoa rainfed seeds (AAX83263 orthologous in *Triticum aestivum*) under redox status alteration ^52^. These and our findings highlight the potential role of plant chitinases in alleviating the effects of different stressors, not only playing protective activities against pathogens but also becoming promising tools for plant engineering abiotic stress mitigation or drought stress biomarkers.

## Conclusions and future perspectives

**Figure 7:**
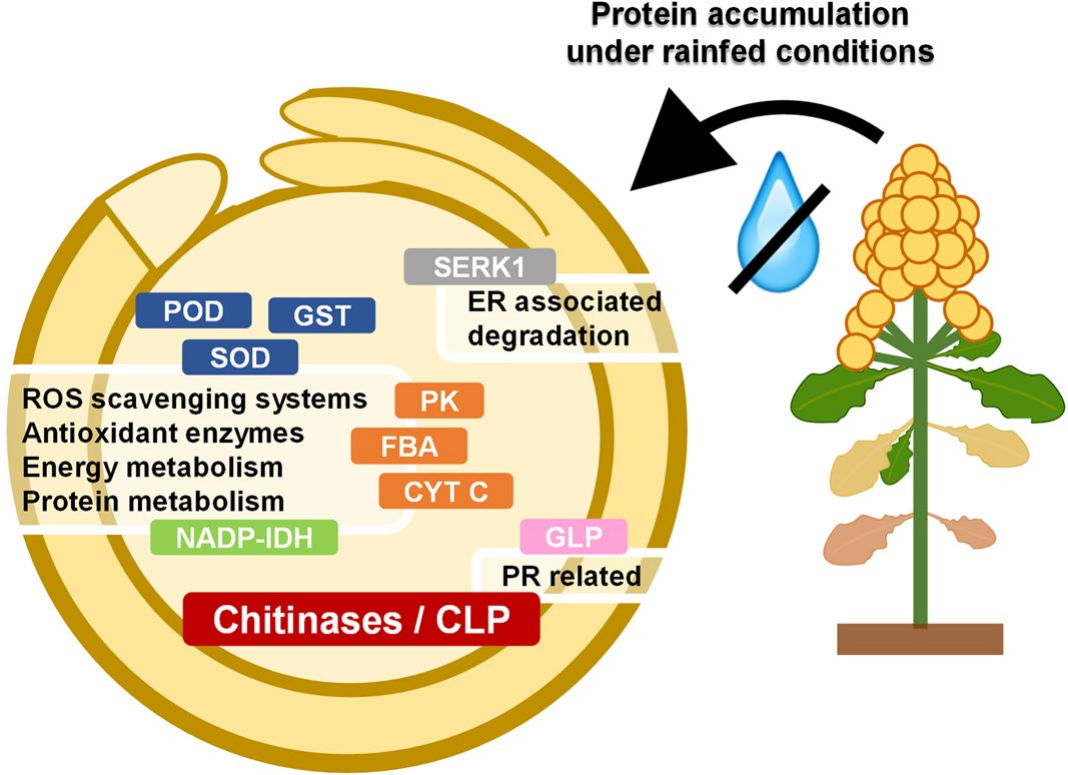
Schematic representation of the main proteins accumulated under rainfed conditions and their GO biological functions. Among the proteins accumulated under rainfed compared to irrigated conditions, there are proteins with functional GO annotations regarding POD: peroxidases (AUR62044027-RA, AUR62003342-RA, AUR62024052-RA, AUR62003343-RA, AUR62026666-RA, AUR62012343-RA, AUR62009723-RA); SOD: superoxide dismutase (AUR62000929-RA); GST: glutathione S-transferase (AUR62008599-RA); FBA: fructose-bisphosphate aldolases 3 (AUR62033531-RA, AUR62028580-RA); CYT C: cytochrome C (AUR62027049–RA, AUR62027048-RA); PK: plastidial pyruvate kinase 2 (AUR62021072-RA); NADP-IDH: NADP isocitrate dehydrogenase (AUR62002238-RA); SERK1: somatic embryogenesis receptor kinase 1 (AUR62018453-RA); GLP: germin-like protein (AUR62037551-RA); and the chitinases (AUR62021380-RA, AUR62021381-RA, AUR62027403-RA, AUR62023849-RA, AUR62002379-RA, AUR62031322-RA, AUR62027403-RA, AUR62023809-RA, AUR62003220-RA), including the two highly accumulated and exclusive chitinases/CLP (chitinase-like proteins) found in seeds under rainfed conditions (AUR62023849-RA and AUR62021380-RA).

Proteomics is considered the most accurate and efficient omic approach, over genomic and transcriptomic studies, since proteins are directly involved in plant phenotypic responses to environmental cues (reviewed in ^75^). These phenotypic responses are modulated by various proteoforms, including protein isoforms and posttranslational modifications (PTMs), which cannot simply infer from transcriptomic data (reviewed in ^76^). In this study, the impact of two contrasting water regimes (rainfed and irrigated conditions) in the field on *C. quinoa* Willd. seed proteomics was evaluated. A total of 2577 proteins were identified resulting in the most complete quinoa seed proteome published to date, highlighting the presence of characteristic seed proteins also found in other plant species such as LEA proteins, oleosins, or SSPs as albumins or globulins, including the quinoa-specific 11S globulin chenopodin ^42^. Moreover, exclusive proteins for each water condition represented a low percentage of the total proteins identified. Proteins that appear with different abundance between water samples were analysed to unravel differences between water treatments. GO terms associated with the differentially abundant proteins in each water condition revealed variations in protein functions, including the high accumulation of proteins involved in catalytic processes under rainfed conditions (Fig. 7). Among these interesting proteins, we found 9 chitinase-related proteins that were significantly more abundant under limiting water availability. These proteins are well-characterized pathogenesis-related (PR) proteins that act degrading chitin in different organisms including plants, animals, or bacteria ^29^. Nonetheless, previous works have shown an induced chitinase activity or the upregulation of chitinase-related gene expression in many plants (including crops) when subjected to various abiotic stresses ^29,64,66–73,77^. Indeed, chitinases represent a huge family of proteins in plants, that include a great number of gene copies and evolutionary divergent sequences that have allowed them to acquire new functionalities resulting in emerging chitinase-like proteins (CLPs) that possess the ability to catalyse or bind different molecules other than chitin ^33^. In the present study, we described 9 chitinase-related proteins in quinoa seeds in response to drought stress. Two of them appeared exclusively in seeds harvested from rainfed conditions. Therefore, these findings could help improve our understanding regarding quinoa strategies that may contribute to improving its adaptation and survival under drought and, possibly, to other abiotic stresses. Moreover, the results here presented open the possibility of utilizing these proteins as plant stress biomarkers for quinoa seeds.

## Materials and methods

### Plant material and experimental conditions

Quinoa (*Chenopodium quinoa* Willd.) seeds from F14 cultivar were kindly provided by Algosur SA (Seville, Spain). Quinoa F14 plants were grown in the field under two environmental conditions at two experimental stations belonging to the *Center for* *Scientific and Technological Research of Extremadura* (CICYTEX, Extremadura, Spain): under irrigated conditions (by applying drip irrigation) (latitude 38° 51′10″ N; longitude 6° 39′10″ W) and under rainfed conditions (latitude 38° 23′ 29″ N; longitude 5° 42′ 28″ W). Both locations were nearly located, and their monthly mean temperatures and precipitations were similar (Supplementary Fig. S1). The total water input under rainfed conditions was 89.3 mm (based on the total rainfall recorded, Supplementary Fig. S1) and 222.7 mm in irrigated conditions (considering 125 mm of waster supplied, quantified by a flow meter in the experimental site, and the total rainfall registered, 96.7 mm, Supplementary Fig. S1). All plant studies were carried out in accordance with relevant institutional, national, or international guidelines and regulations.

Sowing was conducted in February 2019 at a dose of 6 kg ha^−1^ using a mechanical plot drill. Harvesting was conducted at the physiological maturity of the plants. The sampling area was 3 m^2^ per elemental plot. Plants were manually cut at ground level and the seeds were separated using a stationary thresher (Wintersteiger LD 352, Ried, Austria).

### Protein extraction and quantitative label-free proteomic analysis (LC–MS/MS)

#### Protein precipitation

Three biologically independent pools of quinoa seeds obtained from rainfed and irrigated conditions were dried and milled. Fifty mg were solubilized in urea 8 M and filtrated to obtain 1 ml of solubilized protein suspension for each sample before starting the precipitation protocol. Proteins were precipitated by adding cold chloroform/methanol 1/3 (v/v) to each sample, followed by 10 min vortex at 4 °C, and the addition of 3 volumes of Milli-Q water. Later, samples were incubated for 10 min at 4 °C and centrifuged at 1000 g for 2 min to discard the supernatant. To solubilize the precipitated proteins, 3 volumes of methanol were added and mixed by vortex for 10 min. Then, samples were centrifuged at 10,000 g for 5 min. Supernatants were discarded and proteins were resuspended in 2 ml urea 8 M.

#### Protein concentration and trypsin digestion

First, 50 µl of each protein sample (with, approximately, 50 µg of total protein that were quantified by Bradford assay ^78^) were loaded on a 10% acrylamide gel using a Mini-PROTEAN Tetra Cell (Bio-Rad) in order to perform *in gel* protein extract purification. Protein electrophoresis was performed in Laemmli buffer ^79^ at 100 V allowing the sample to run for 35 min (2 cm) into the stacking gel). Then, gels were fixed in methanol 50% (v/v) and phosphoric acid 2% (v/v) for 30 min and then washed, rinsing the gel twice with Milli-Q water. Later, gels were incubated in methanol 33% (v/v), ammonium sulphate 17% (v/v) and phosphoric acid 3% (v/v) for 45 min. Protein bands were visualized after incubating the gel in colloidal Coomassie (G-250) and methanol (6.6 mg/ml) overnight and rinsing the excess of Coomassie solution with Milli-Q water.

The remains of Coomassie solution were removed by rinsing protein gels twice with pure acetonitrile (ACN) and ammonium bicarbonate 25 mM. Disulphide bonds were reduced using dithiothreitol (DTT) 20 mM in ammonium bicarbonate 25 mM, 56 °C, for 30 min and then blocked with iodoacetamide 22.5 mM in ammonium bicarbonate 25 mM, 15 min, in darkness. Two more washes were performed with ACN before completing dehydration of the gel using the SpeedVac (Thermo Fisher Scientific, Massachusetts, United States) for 30 min.

Finally, protein bands (obtaining a single protein band per sample) were cut, and trypsin (Roche, Mannheim, Germany) 1:100 (v/v) in ammonium bicarbonate 25 mM was added for digestion at 37 °C overnight. Digested peptides were recovered from the supernatant and dried using the SpeedVac (Thermo Fisher Scientific, Massachusetts, United States) for 30 min and resuspended in 31 µL ACN 2% (v/v) and formic acid 0.1% (v/v). One µL of each protein extraction was used to determine sample concentration using Invitrogen™ Qubit™ 3 (Thermo Fisher Scientific, Massachusetts, United States).

#### Reversed-phase liquid chromatography (LC) for peptide separation

One µg of each protein extraction was injected into a nano-HPLC Easy-nLC 1000 (Thermo Fisher Scientific, Massachusetts, United States). First, samples were concentrated using a precolumn PEPMAP100 C18 NanoViper Trap (Thermo Fisher Scientific, Massachusetts, United States). Then, samples were separated through a 50 cm column PEPMAP RSLC C18 (Thermo Fisher Scientific, Massachusetts, United States) on a gradient of ACN 5% to 40% (v/v) and formic acid 0.1% (v/v) for 120 min.

#### Data-dependent acquisition (DDA) for shotgun proteomics

Peptide fractions were electrospray ionized in positive mode and analyzed by a quadrupole Orbitrap mass spectrometer Q Exactive HF (Thermo Fisher Scientific, Massachusetts, United States) in DDA mode. From each mass spectrometry (MS) scan (between 390 and 1700 Da), the 15 most intense precursors (charged between 2 + and 5 +) were selected for their high collision energy dissociation (HCD) fragmentation. Then, the corresponding tandem mass spectrometry (MS/MS) spectra was acquired.

### Quantitative proteomic analysis

#### Protein identification

Data generated by LC–MS/MS for each quinoa seed sample was analyzed using Proteome Discoverer 2.4 (Thermo Fisher Scientific, Massachusetts, United States). Each MS/MS spectra was identified by peptide-spectrum matches (PSMs) comparing them to theoretical masses obtained from the original precursor mass fragmentation, using *Chenopodium quinoa* proteome annotation in UniProt (https://www.uniprot.org/proteomes?query=(organism_id:63459), a curated version of JGI Phytozome database (https://phytozome-next.jgi.doe.gov/; Phytozome genome ID: 392), taxonomically restricted to *Chenopodium quinoa* v1.0. Identified peptides were assigned to the annotated *C. quinoa* proteins. Whether a peptide may be assigned to different proteins, the software used the parsimony principle to generate a master protein. The Percolator algorithm was used to estimate the false discovery rate (FDR). High-confidence proteins were identified and filtered by *p adjusted value (p-adj)* < *0.05*.

The mass spectrometry proteomics data have been deposited to the ProteomeXchange Consortium via the PRIDE ^80^ partner repository with the dataset identifier PXD038953 and https://doi.org/10.6019/PXD038953.

#### Peptide and protein normalization

Proteome Discoverer 2.4 (Thermo Fisher Scientific, Massachusetts, United States) was used to determine peptide and protein abundance. First, mass recalibration was performed with Sequest HT comparing database and identified proteins, getting a chromatography alignment of the samples with a tolerance of up to 10 min. Then, an alignment of the retention time of all samples was performed to quantify precursor ions (considering unique peptides that were present in, at least, two of the three replicates). Finally, the total protein amount was normalized among samples using peptide total abundance. Also, Proteome Discoverer 2.4 (Thermo Fisher Scientific, Massachusetts, United States) was used to represent the Principal Component Analysis (PCA) and the heat map with the common and exclusive proteins found in each biological replicate of the seeds harvested from irrigated and rainfed conditions (Supplementary Figures S2 and S3).

#### Sample pooling and relative protein quantification

Three biological replicates were analyzed for each treatment (irrigated and rainfed conditions) using a No Nested/Pairwise design. Protein quantification values were obtained from peptide ratios calculated as a geometric median of the peptide ratio in each biological replicate (Supplementary Table S1).

To compare the variation in protein abundance between the two water conditions, the log2 Fold Change (log2FC) ratio protein abundance of rainfed versus irrigated conditions was calculated. A threshold of log2FC ≥ 1 or log2FC ≤ − 1 was used to determine rainfed and irrigated protein differential enrichment, respectively. In addition, an analysis of variance (ANOVA) was performed to estimate statistically significant enriched proteins between quinoa seeds harvested from irrigated and rainfed conditions with a significance level of *0.05*. *P-values* obtained with this analysis were corrected (*p-adj*), taking into account the False Discovery Rate (FDR) applying Benjamin&Hochberg (BH) test (Supplementary Table S3 and Table S4).

#### Gene ontology (GO) enrichment analysis

*C. quinoa* Willd accession PI 614,886 coding sequences (CDSs) from JGI’s last annotation version comes from 2017. To improve gene ontology (GO) terms associated with each protein, a new functional reannotation was carried out in our laboratory as follows. Protein sequences were downloaded and blasted against NCBI non-redundant (nr) database (January 2022) using DIAMOND ^81^. Then, BLAST output was processed with Blast2GO software (https://www.blast2go.com) ^82^ to get a tabular file with the corresponding updated functional annotation including GO terms. Functional enrichment was studied in different groups: proteins that appeared exclusively in seeds from plants grown under irrigated or rainfed conditions; proteins that were enriched in seeds from plants grown under rainfed conditions (log_2_FC ≥ 1, *p-adj* ≤ *0.05*; n = 3) and proteins enriched in seeds from plants grown under irrigated conditions (log_2_FC ≤  − 1, *p-adj* ≤ *0.05*; n = 3). For each group of proteins, a GO term enrichment analysis was performed in R ^83^ using as annotation the list of terms obtained with Blast2GO using the topGO package ^84^.

### Phylogenetic analysis

Phylogenetic analyses were performed with protein sequences using the online platform NGPhylogeny.fr ^85^, following the FastTree/OneClick workflow (https://ngphylogeny.fr/): MAFFT 7.407 for multiple alignment ^86^, BMGE 1.12_1 for alignment curation ^87^, FastTree 2.1.11 for approximately maximum likelihood phylogenetic tree inference ^88^ and Newick Display 1.6 for tree rendering ^89^.

### Domain prediction, protein representations and sequence alignment

Protein–protein BLAST (BLASTp-NCBI; https://blast.ncbi.nlm.nih.gov/Blast.cgi) using clustered nr database (nr database clustered at 90% identity and 90% sequence length) was performed to deepen into chitinase-related proteins. NCBI Batch Web CD-search tool (https://www.ncbi.nlm.nih.gov/Structure/bwrpsb/bwrpsb.cgi) was used for protein domain prediction. FASTA sequences for each protein were uploaded to Batch CD-search using automatic search mode which directly launched live search as sequences were submitted explicitly in FASTA format. CD database (CDD) was selected to perform the search, setting 0.01 as the statistical significance threshold (*E-value*). Protter (http://wlab.ethz.ch/protter/start/) was used as a plotting tool to graphically represent proteins ^90^. Multiple sequence alignment by CLUSTALW (https://www.genome.jp/tools-bin/clustalw) was performed to compare protein homology.

### Ethical approval and informed consent

Javier Matías (JM) and Verónica Cruz (VC) identified the seed samples harvested from the field that were used in the current study. The quinoa seeds used in the current study were kindly provided by the company Algosur SA (Seville, Spain).

## Supplementary Information

Supplementary Tables.

Supplementary Figures.

## Data Availability

Raw data from the shotgun proteomic experiment is publicly available at ProteomeXchange Consortium via the PRIDE ^80^ partner repository with the Project Name: Shotgun proteomics of quinoa seeds from plants grown under irrigated and rainfed conditions, dataset identifier PXD038953, https://doi.org/10.6019/PXD038953. Derived data supporting the findings of this study are available within the article and its supplementary materials published online. The quinoa seeds used to carry out this study are available upon request from the company Algosur SA (Seville, Spain).
