# Supplementary material for: Shotgun proteomics of quinoa seeds reveals chitinases enrichment under rainfed conditions
Source: Sci Rep. 2023 Mar 27;13:4951. doi: 10.1038/s41598-023-32114-5 (PMC10043034; doi:10.1038/s41598-023-32114-5)
Supplement: Supplementary file 2 — Supplementary Figures. [file 41598_2023_32114_MOESM2_ESM.pptx]

## Slide 1
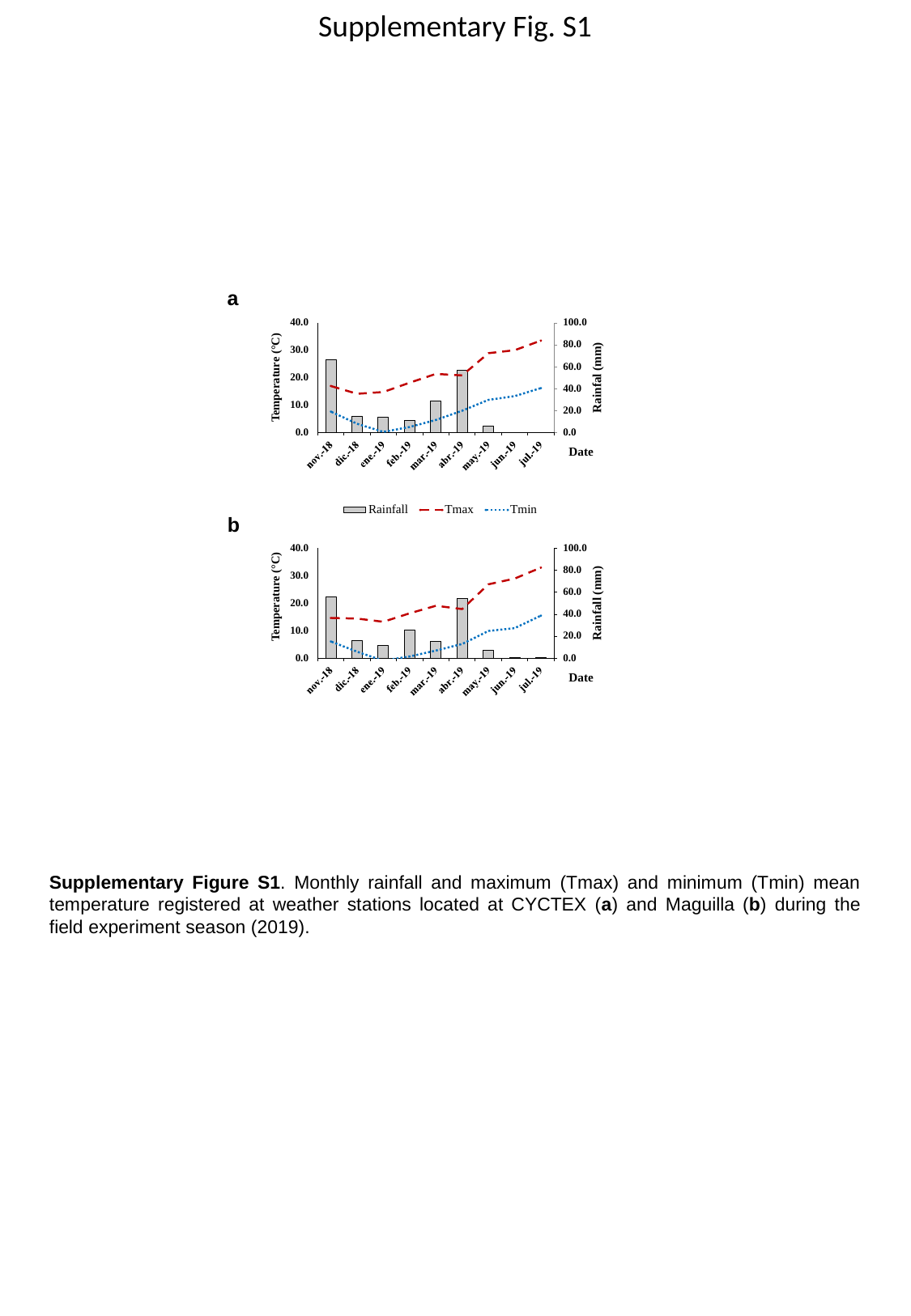

Supplementary Fig. S1
a
Temperature (°C)
b
Temperature (°C)
Supplementary Figure S1. Monthly rainfall and maximum (Tmax) and minimum (Tmin) mean temperature registered at weather stations located at CYCTEX (a) and Maguilla (b) during the field experiment season (2019).

## Slide 2
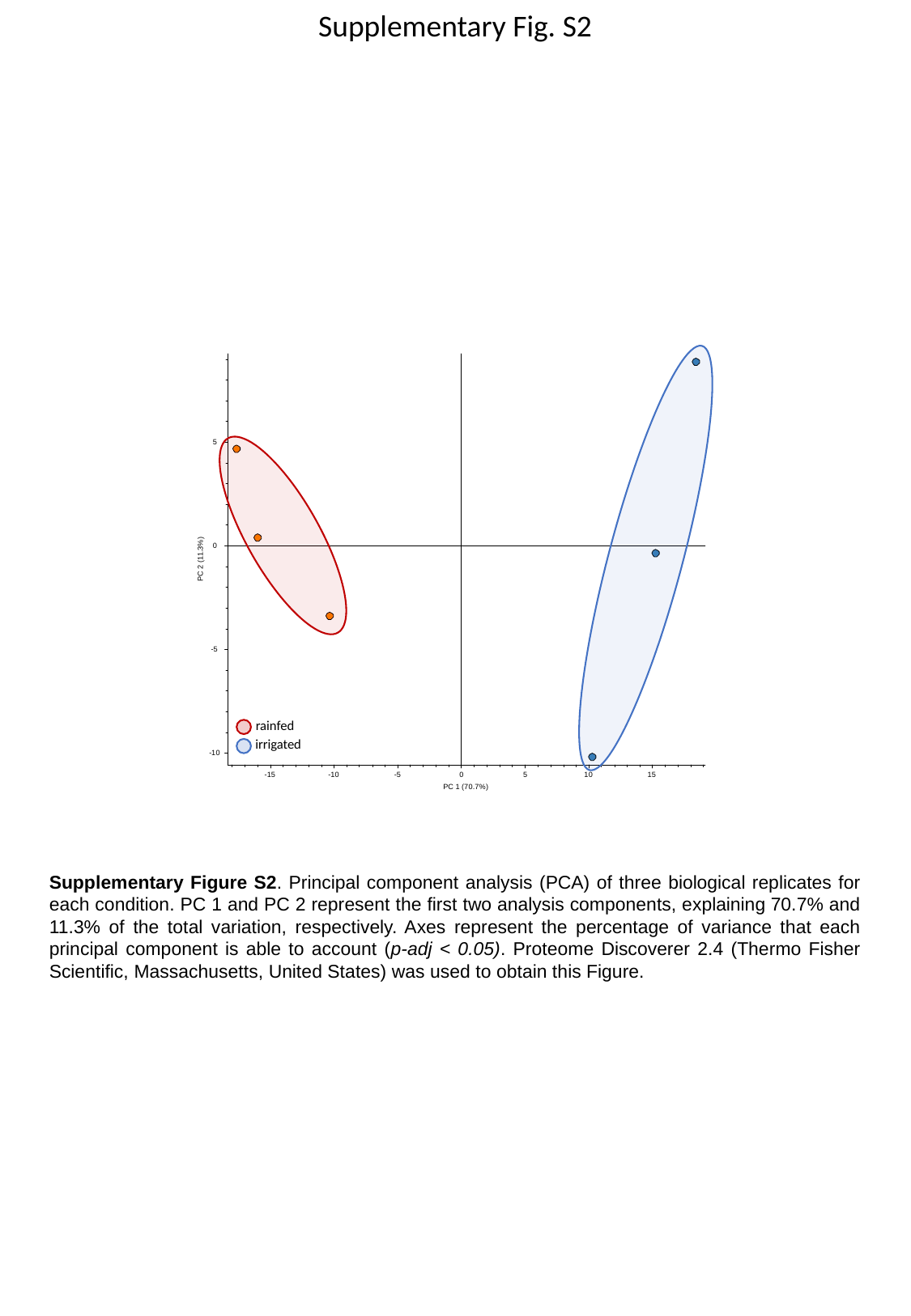

Supplementary Fig. S2
rainfed
irrigated
Supplementary Figure S2. Principal component analysis (PCA) of three biological replicates for each condition. PC 1 and PC 2 represent the first two analysis components, explaining 70.7% and 11.3% of the total variation, respectively. Axes represent the percentage of variance that each principal component is able to account (p-adj < 0.05). Proteome Discoverer 2.4 (Thermo Fisher Scientific, Massachusetts, United States) was used to obtain this Figure.

## Slide 3
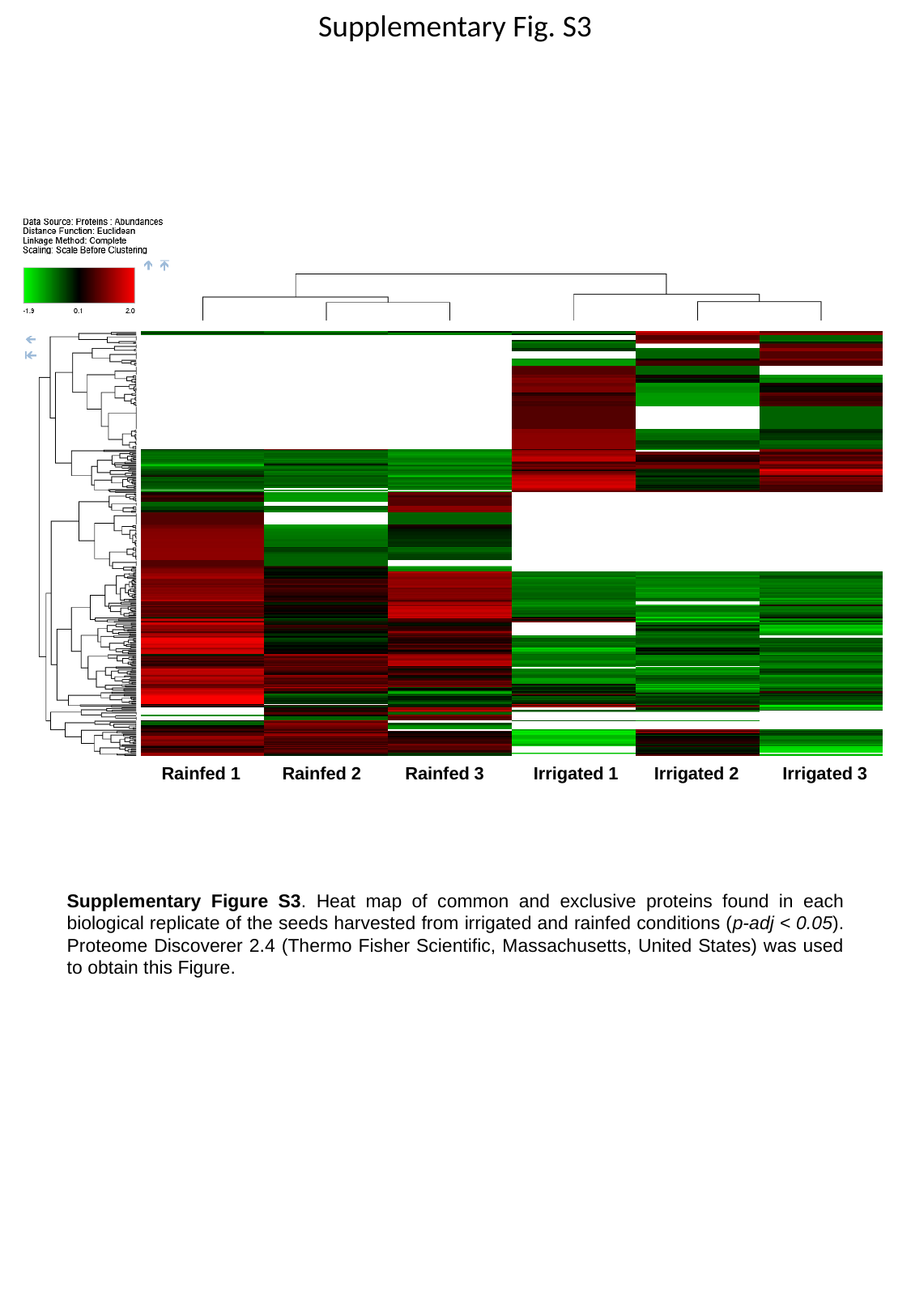

Supplementary Fig. S3
Rainfed 1
Rainfed 2
Rainfed 3
Irrigated 1
Irrigated 2
Irrigated 3
Supplementary Figure S3. Heat map of common and exclusive proteins found in each biological replicate of the seeds harvested from irrigated and rainfed conditions (p-adj < 0.05). Proteome Discoverer 2.4 (Thermo Fisher Scientific, Massachusetts, United States) was used to obtain this Figure.

## Slide 4
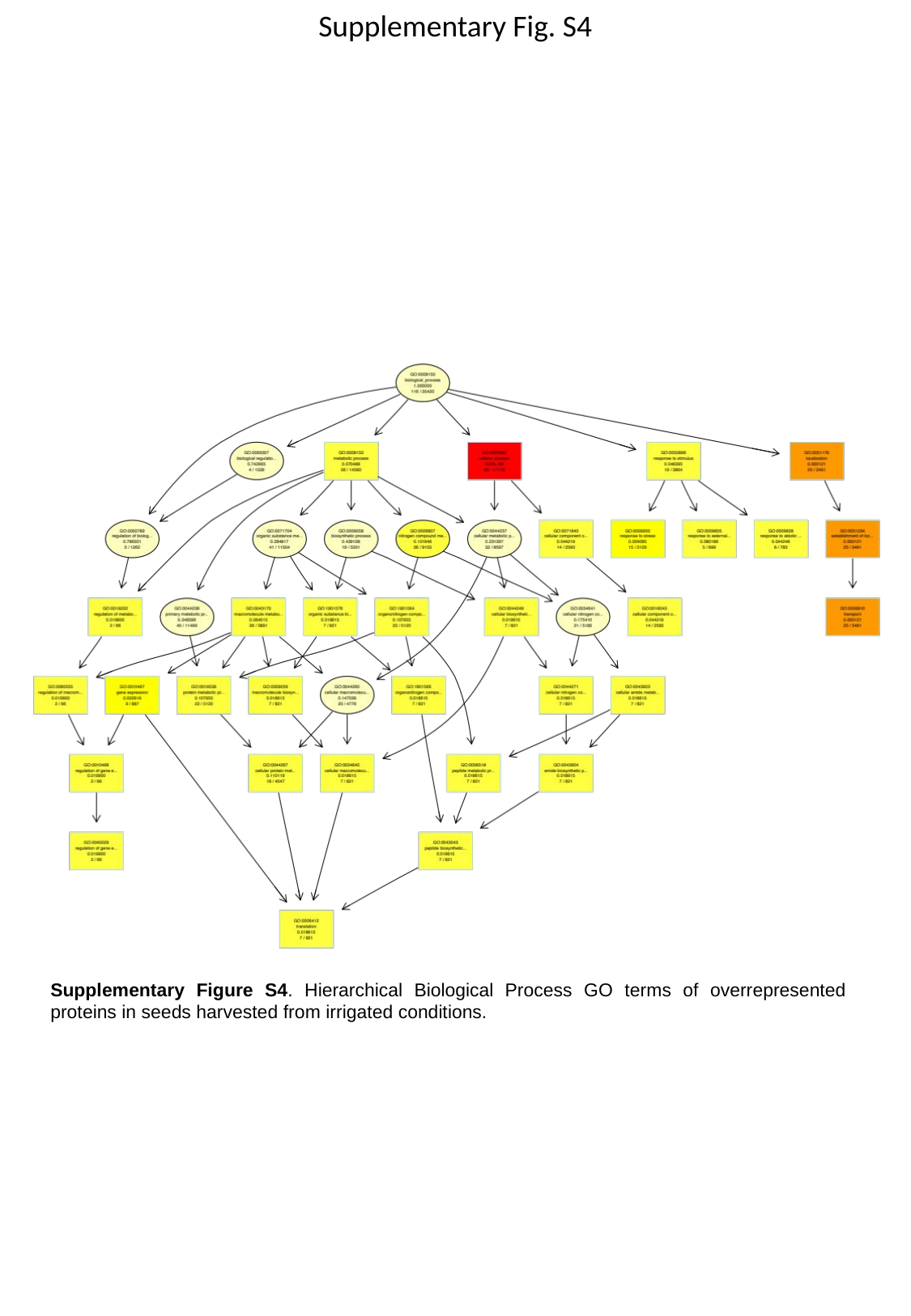

Supplementary Fig. S4
Supplementary Figure S4. Hierarchical Biological Process GO terms of overrepresented proteins in seeds harvested from irrigated conditions.

## Slide 5
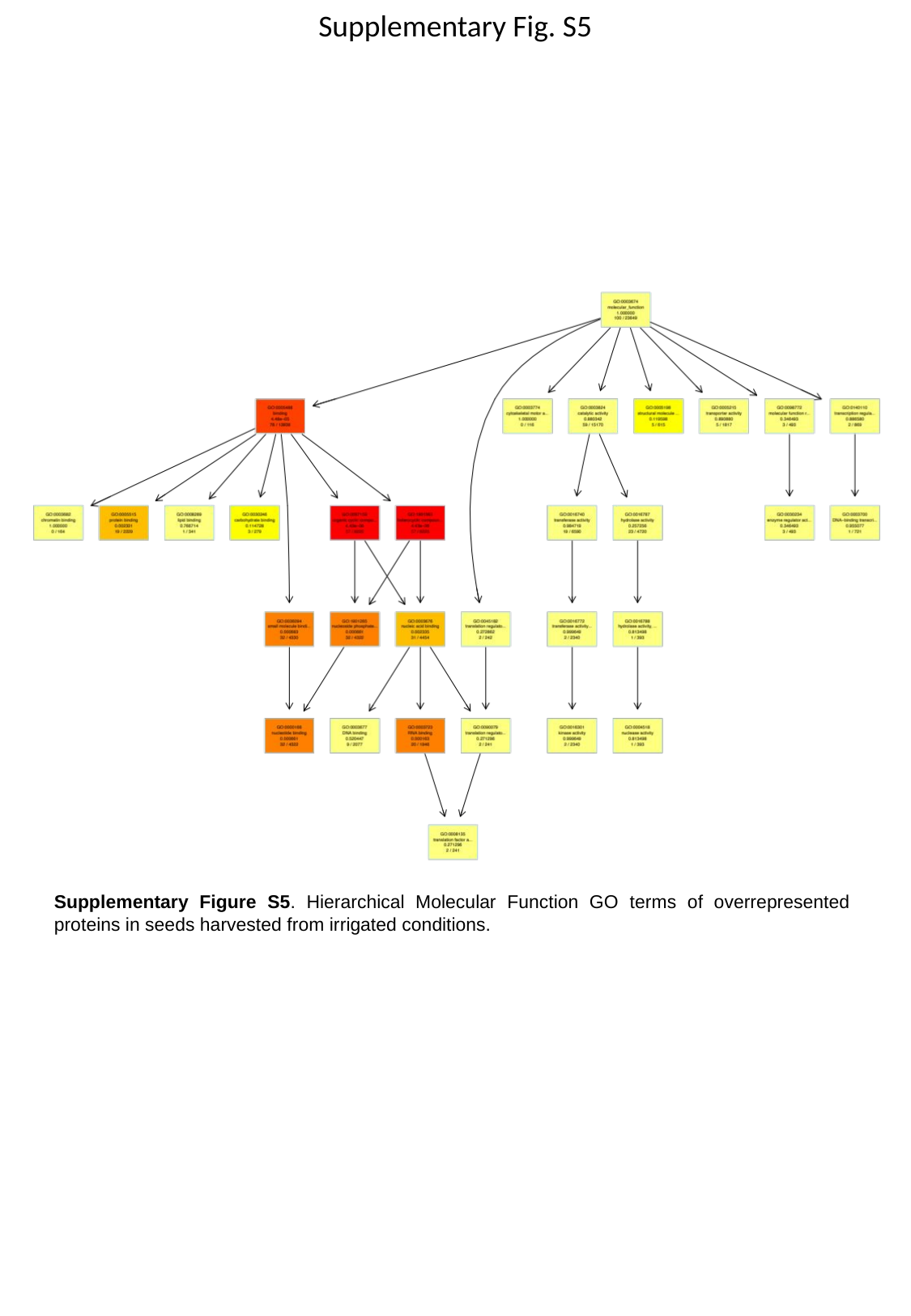

Supplementary Fig. S5
Supplementary Figure S5. Hierarchical Molecular Function GO terms of overrepresented proteins in seeds harvested from irrigated conditions.

## Slide 6
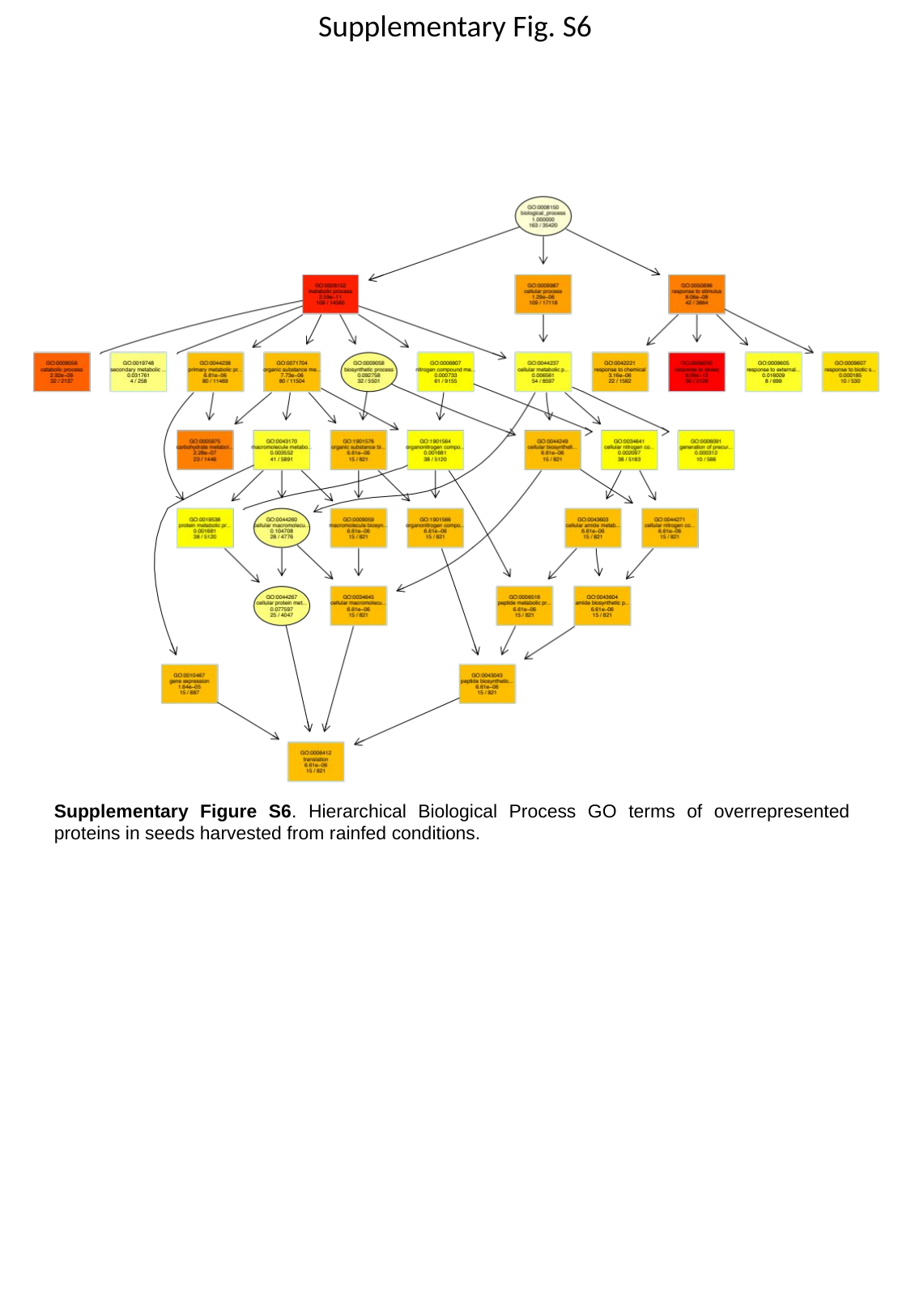

Supplementary Fig. S6
Supplementary Figure S6. Hierarchical Biological Process GO terms of overrepresented proteins in seeds harvested from rainfed conditions.

## Slide 7
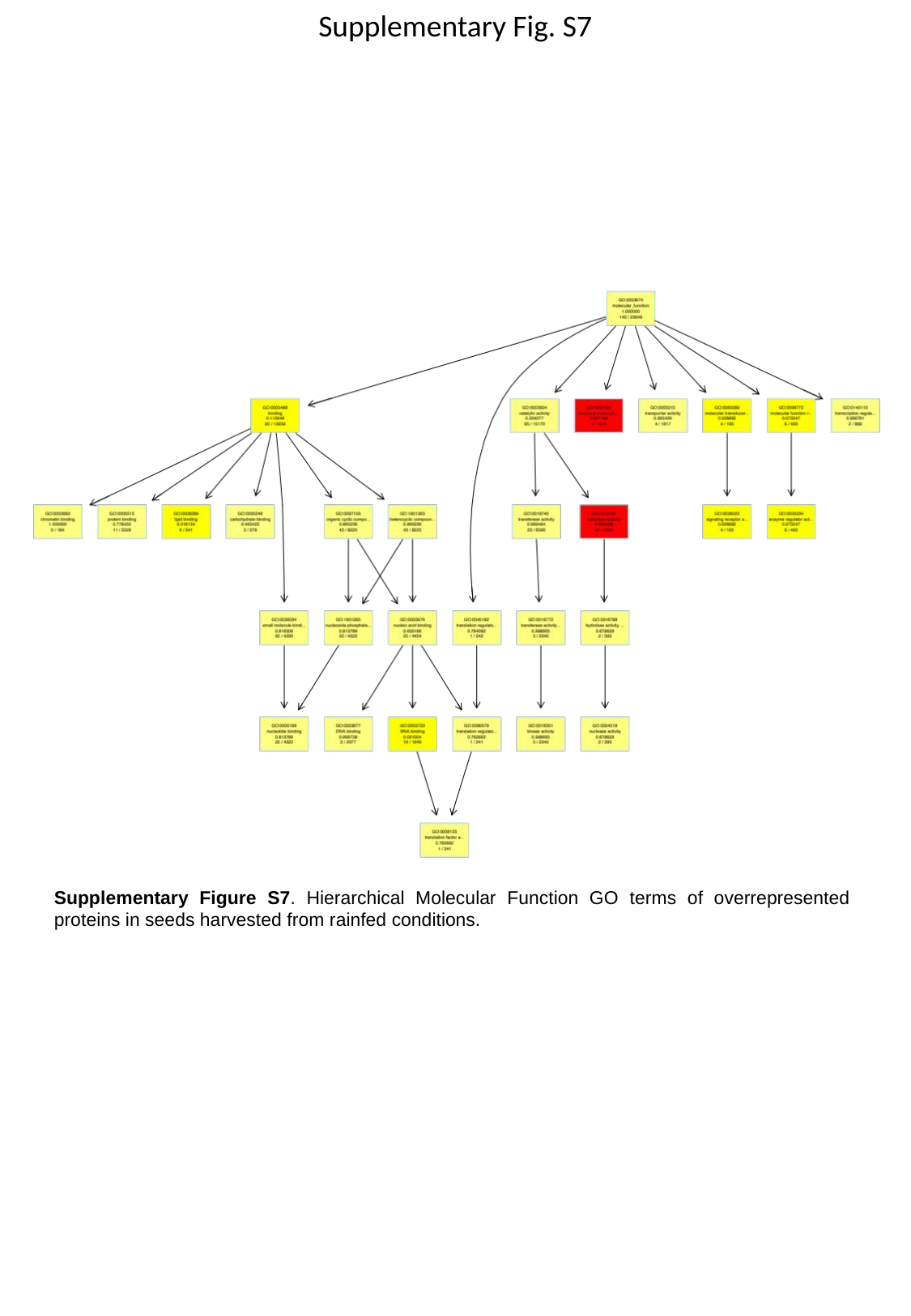

Supplementary Fig. S7
Supplementary Figure S7. Hierarchical Molecular Function GO terms of overrepresented proteins in seeds harvested from rainfed conditions.

## Slide 8
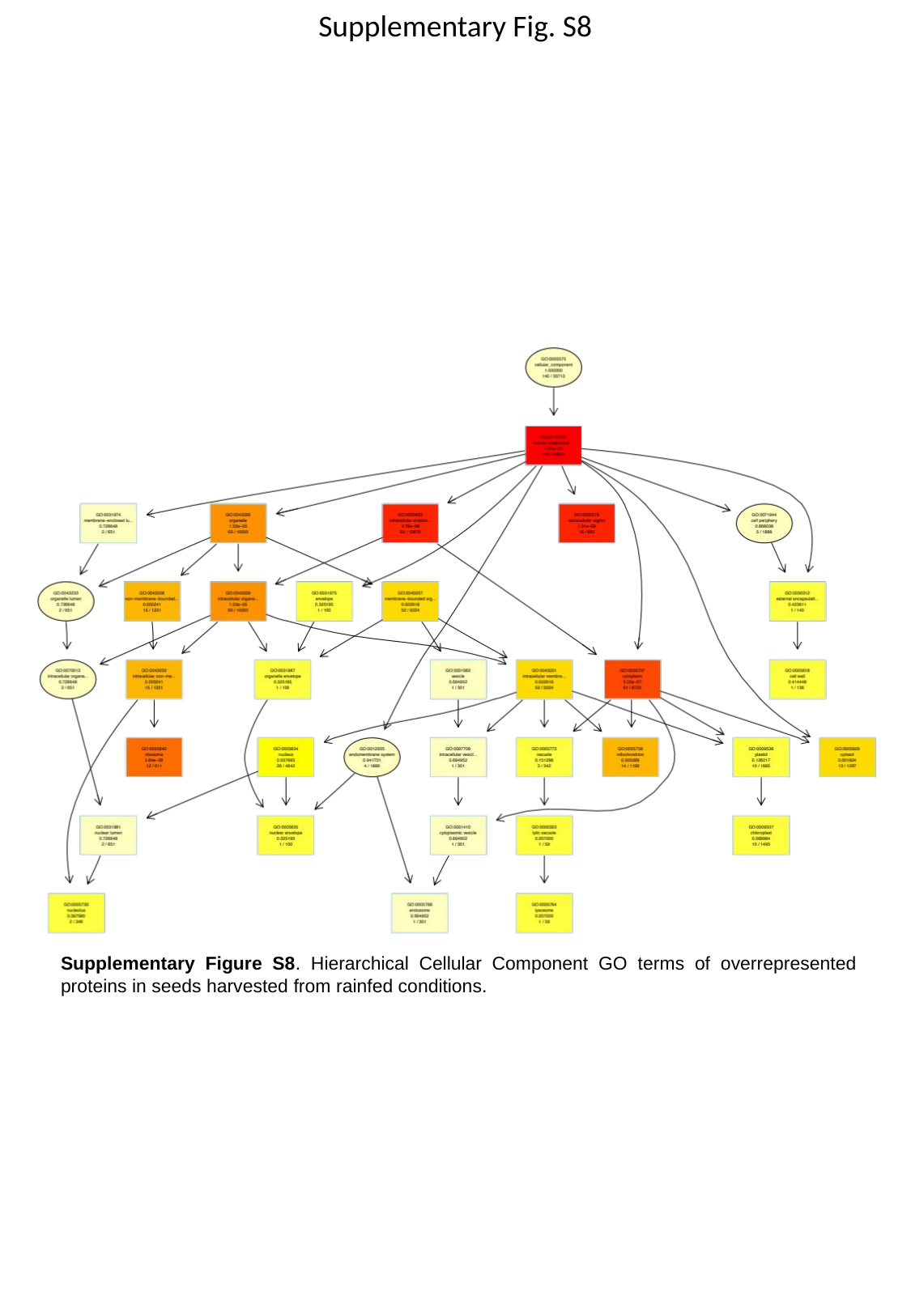

Supplementary Fig. S8
Supplementary Figure S8. Hierarchical Cellular Component GO terms of overrepresented proteins in seeds harvested from rainfed conditions.

## Slide 9
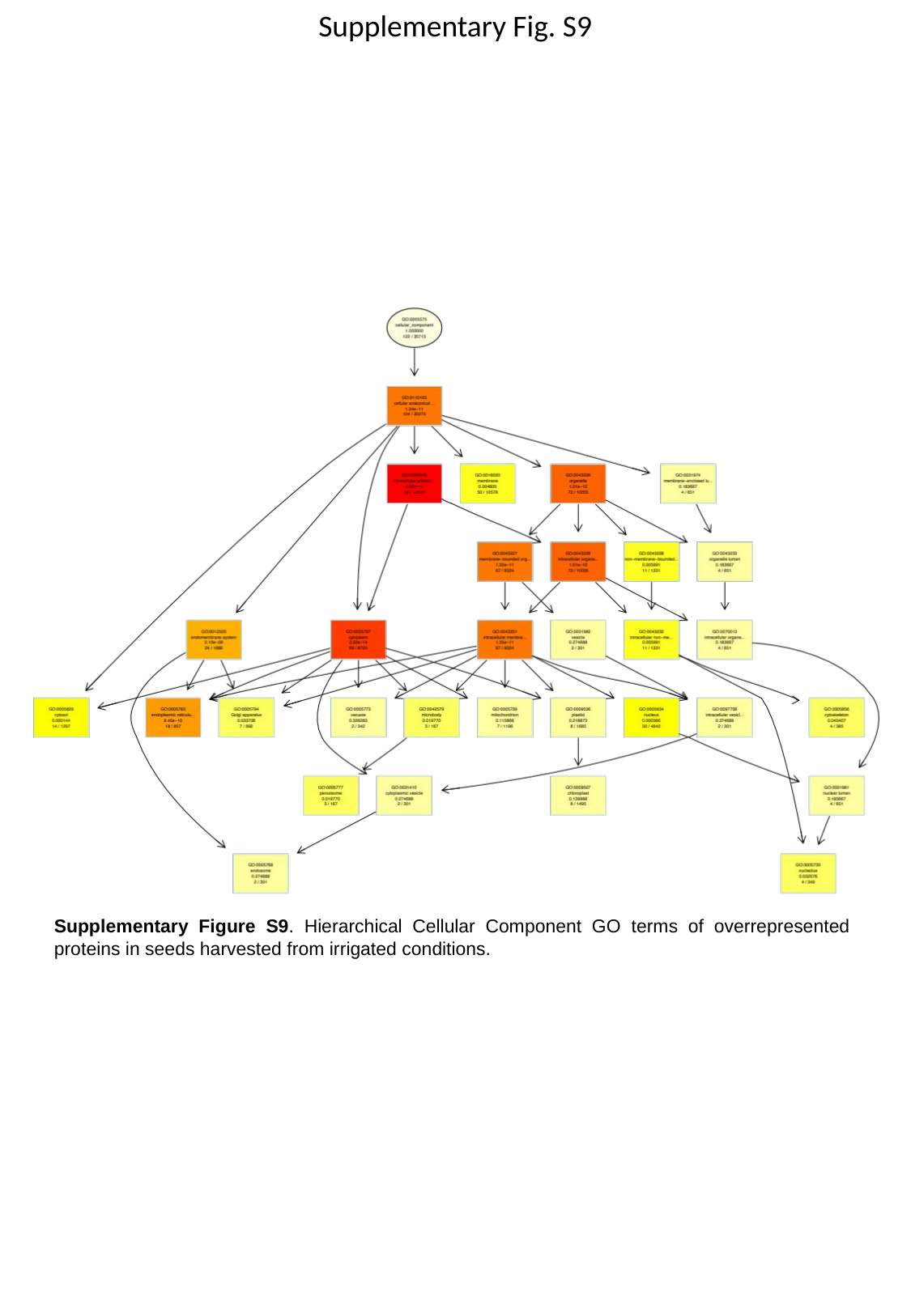

Supplementary Fig. S9
Supplementary Figure S9. Hierarchical Cellular Component GO terms of overrepresented proteins in seeds harvested from irrigated conditions.
